# Emerging *p*‐Block Metal‐Based Electrocatalysts for Energy Conversion

**DOI:** 10.1002/smll.202514932

**Published:** 2026-03-22

**Authors:** Jack Jon Hinsch, Jinqiu Hu, Li Wang, Yun Chen, Huai Qin Fu, Zhenzhen Wu, Mengqing Hu, Mengyang Dong, Porun Liu, Lei Zhang, Yun Wang, Zhonghong Xia, Liang Wang

**Affiliations:** ^1^ Centre for Catalysis and Clean Energy School of Environment and Science Griffith University Gold Coast Queensland Australia; ^2^ College of Materials Science and Engineering Donghua University Shanghai China; ^3^ School of Engineering and Built Environment Griffith University Gold Coast Queensland Australia; ^4^ Institute for Sustainable Energy College of Sciences Shanghai University Shanghai China

**Keywords:** alloy design, CO_2_ reduction, electrocatalysis, nitrogen reduction, oxygen reduction, p‐block metals, single‐atom catalysts

## Abstract

P‐block metal‐based catalysts are an emerging, effective, and sustainable alternative to precious metal electrocatalysts for energy conversion. These p‐block‐based materials exhibit unique characteristics, such as oxophilicity, weak hydrogen adsorption, and flexible electronic tuning, which rival those of Pt‐group metals. Several morphologies (single‐atom catalysts, pure metals, alloys, compounds, and doped systems) have achieved remarkable catalytic efficiencies and selectivity utilizing p‐block metals. Recent advances have focused on tailoring the electronic band, surface morphology, and coordination environments, significantly enhancing catalytic stability and activity. Mechanistic studies highlight deviations from traditional d‐band scaling relationships, offering novel design strategies for reaction‐specific optimization. However, challenges remain. Achieving industrially relevant current densities, long‐term stability, and scalable synthesis remains troublesome. This review synthesizes recent progress in p‐block metal‐based electrocatalysts across various morphologies to identify performance and mechanistic trends for energy conversion applications (e.g., oxygen reduction, nitrate reduction, and carbon dioxide reduction reactions). Critical research directions are identified, and fundamental gaps in p‐block metal research are discussed. By establishing the core understanding, future research can focus on untapping the potential of p‐block metals for next‐generation electrocatalysts.

## Graphical abstract

P‐block metal‐based catalysts have emerged as efficient and sustainable alternatives to precious metal electrocatalysts for energy conversion. This review comprehensively surveys diverse *p*‐block metal‐based architectures—including anisotropic and homogeneous systems—and systematically elucidates their roles in key electrocatalytic reactions, such as CO_2_RR, NRR, and ORR, highlighting fundamental principles, recent advances, and future outlooks.

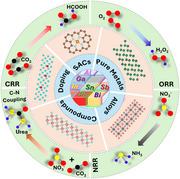

## 1 Introduction

Our society relies heavily on the supply of non‐renewable sources, including natural gas, coal, and oil. However, there is only a finite pool of these resources to extract before a severe energy crisis strikes [1, 2]. Additionally, even essential products like ammonia are primarily derived from natural gas [3]. Addressing these issues requires not only the development of renewable energy systems but also the discovery of efficient, cost‐effective, and durable catalysts capable of facilitating critical chemical transformations.

The development of efficient and sustainable electrocatalysts is central to advancing next‐generation energy conversion technologies, including fuel cells, water electrolysis, and carbon dioxide reduction. An effective catalyst requires specific characteristics to be utilized in industrial fields. To be competitive in industry, a catalyst must be selective, durable, cost‐effective, and efficient [4, 5]. The three reactions that have received the most attention for developing new catalysts are the oxygen reduction, nitrogen reduction, and carbon dioxide reduction reactions (commonly referred to as ORR, NRR, and CO_2_RR, respectively) [6, 7]. Currently, little progress has been made on replacing or improving current industry standards for these reactions. Often, the required catalysts are composed of rare and/or expensive transition metals, which limit the commerciality of alternative solutions to fossil fuels.

The platinum group metals (PGMs) are characterized by their catalytic abilities and use in electrochemical reactions. The PGMs' electronic structure, including the Fermi energy and high density of states, is a key factor toward their catalytic ability [8]. PGMs are also known for their superior physical and chemical stability, allowing them to be involved in many industrial catalytic reactions and stable technologies [9]. Despite the clear advantages these metals hold, their high cost remains a significant barrier to scaling up renewable technologies. Therefore, we must identify a replacement for these transition metals that improves the competitiveness of alternative energy and synthesis technologies. A new material that not only exhibits the ideal electronic properties, but also the durability of the PGMs needs to be discovered.

**FIGURE 1:**
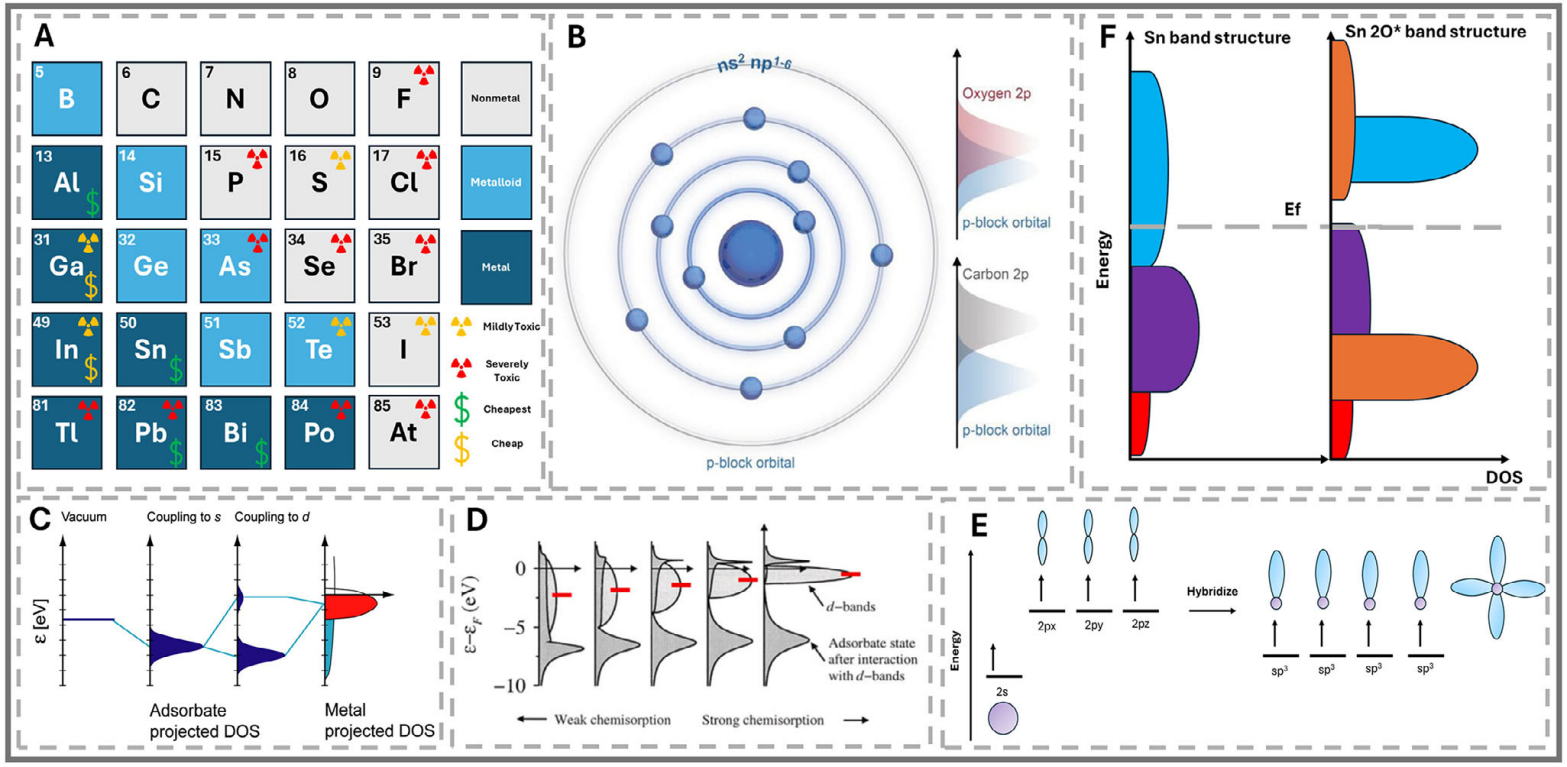
(A) Classification of p‐block elements with toxicity (LDK) and cost relative to Pt. The cost was only evaluated for the p‐block metals of interest. (B) General electronic configurations of p‐block metals and their bonding tendencies with oxygen‐ and carbon‐based compounds. (A,B) Reproduced with permission [14]. Copyright 2023, Wiley‐VCH. (C,D) Principles of metal–adsorbate interactions, including H adsorption on transition metals and the d‐band center model. (C) Reproduced with permission [15]. Copyright 2021, National Academy of Sciences. (D) Reproduced with permission [16]. Copyright 2000, Elsevier Inc. (E) Orbital hybridization diagram illustrating sp^3^ formation. (F) Example of band structure modification in Sn upon oxygen adsorption, showing how p‐block band centers shift under adsorbates.

Among emerging alternatives, p‐block metals, such as aluminum (Al), gallium (Ga), indium (In), tin (Sn), bismuth (Bi), and lead (Pb), have attracted attention due to their earth abundance, low cost, and lower toxicity (in most cases) (see Figure 1A). Characterized by dominant p‐orbital electron configurations (ns^2^np^x^), these elements offer unique catalytic behavior, particularly in reactions involving small, stable molecules like N_2_, O_2_, and CO_2_ (see Figure 1B) [10, 11]. A notable advantage of p‐block metals is their inherent suppression of the competing hydrogen evolution reaction (HER), a common challenge in electrocatalysis [12, 13]. These desirable properties have caused p‐block metals to gain in popularity in the literature.

One key difference between p‐block and traditional metals is the limited oxidation states of the p‐block metals. Therefore, the mechanisms for triggering catalytic activity and designing a better p‐block catalyst differ from those of transition metal‐based catalysts [14, 15, 16]. In conventional d‐block metals, the widely accepted d‐band theory describes how the position of the d‐band center relative to the Fermi level governs the strength of adsorption (see Figure 1C,D) [15, 16]. The adsorption energy and mechanisms govern the reaction rate, energy barriers, and selectivity of the catalysts. However, p‐block metals lack the partially filled d‐block orbitals that define this behavior. For p‐block metals, the alignment of their p‐orbitals and occupancy are strong factors in their catalytic performance [17]. Recent studies have highlighted the significance of the p‐band center, orbital hybridization, and local electronic structure in controlling reaction pathways [18]. Differing p‐block elements changes the degree of sp^2^ or sp^3^ hybridization due to size and electronic configuration. Therefore, the degree of customisation varies greatly and alters the reactivity depending on the level of band structure (see Figure 1E,F) [19]. The unique orbital strucrapidlyows for alternative reaction pathways, offering more flexible control over the adsorption energies of key intermediates compared to the more rigid d‐band governed systems. Therefore, p‐block metal's ability to alter and customize the band structure holds unique potential for future catalyst designs [15]. Research has primarily focused on the local charge, p‐band center, and work function as indicators of strong p‐block catalysts. However, the p‐block metals still suffer from unsatisfactory current densities and durability [20]. Therefore, the p‐block elements (on their own) are utilized as synergistic supports/components for electrocatalytic materials [21]. When incorporated as dopants or single‐atom catalysts, p‐block elements can also exploit p–d orbital hybridization or even mimic d‐band back‐donation effects, breaking conventional scaling relationships that constrain transition metals [22]. Despite growing research interest, there remains no clear consensus on the optimal strategies for employing p‐block metals in catalysis.

**FIGURE 2:**
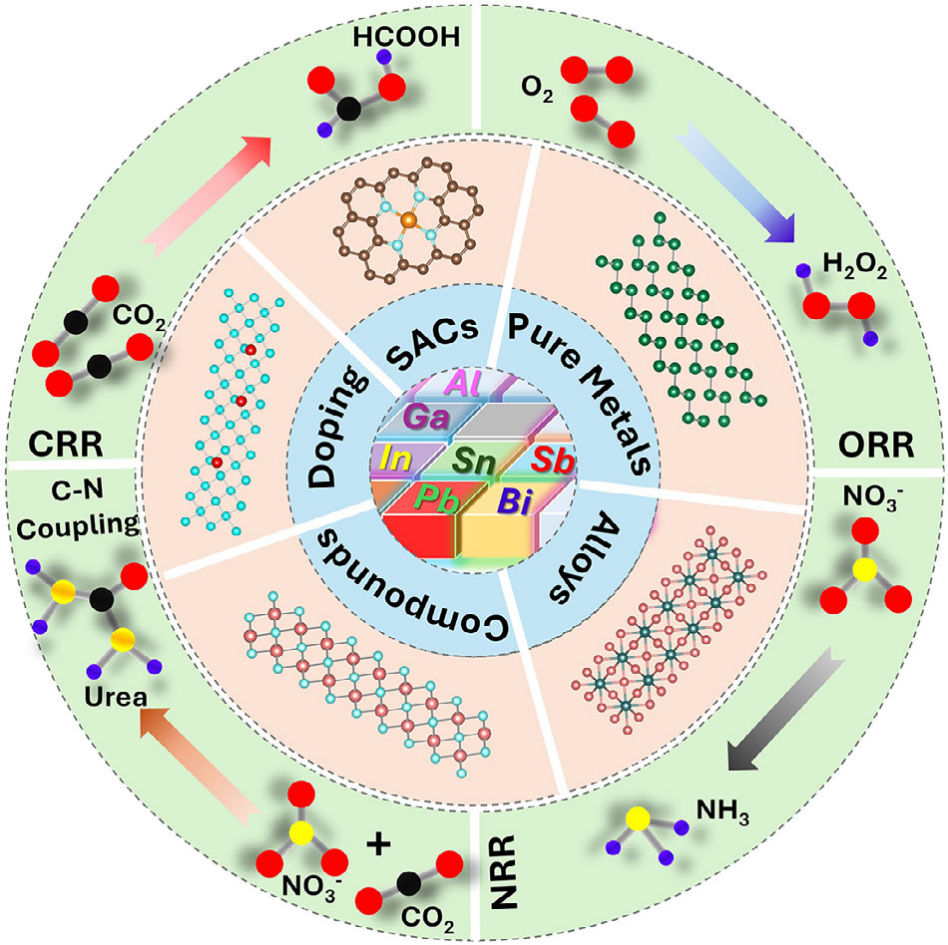
Schematic overview of emerging *p*‐block metal‐based electrocatalysts and their potential applications in energy conversion.

**FIGURE 3:**
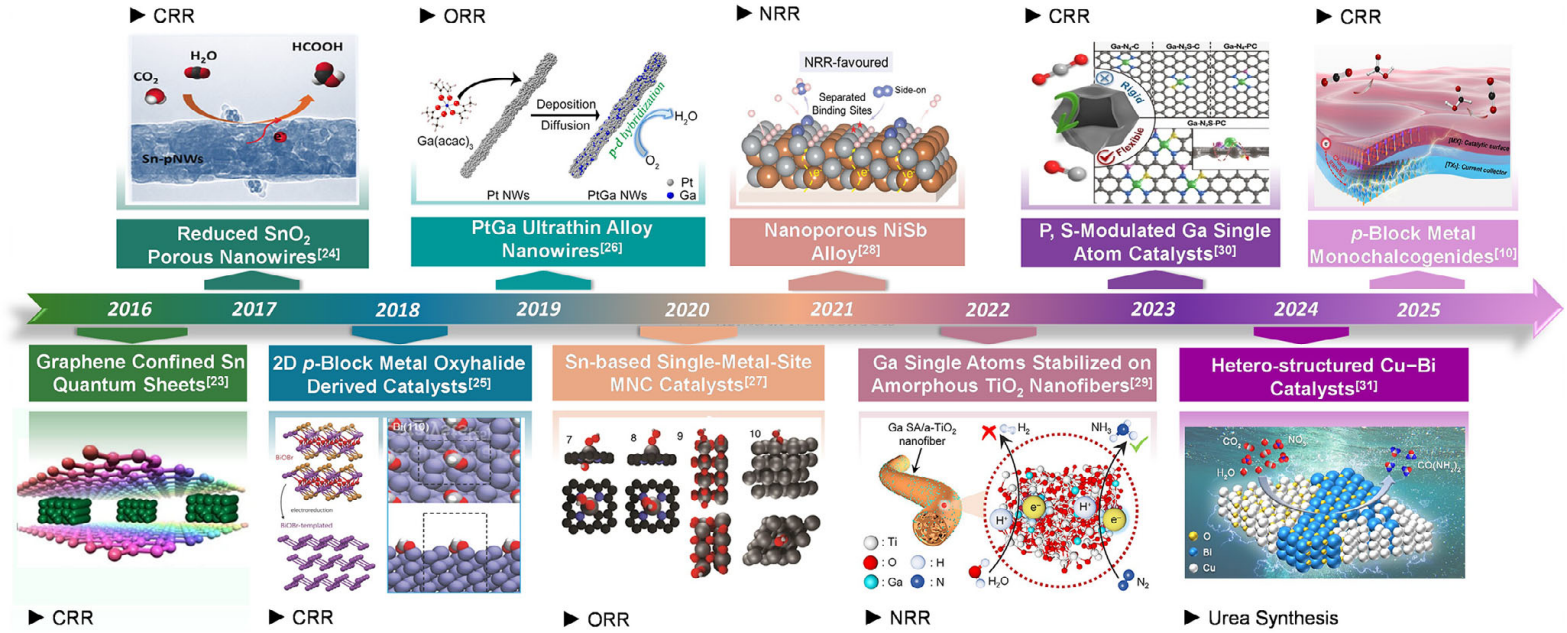
An overview of the key developments in *p*‐block metal‐based electrocatalysts for energy conversion during the past decade [10, 23, 24, 25, 26, 27, 28, 29, 30, 31]. Reproduced with permissions [23]. Copyright 2016, Springer Nature. Reproduced with permissions [24]. Copyright 2017, Wiley‐VCH. Reproduced with permissions [25]. Copyright 2018, Wiley‐VCH. Reproduced with permissions [26]. Copyright 2019, American Chemical Society. Reproduced with permissions [27]. Copyright 2020, Springer Nature. Reproduced with permissions [28]. Copyright 2021, Wiley‐VCH. Reproduced with permissions [29]. Copyright 2022, American Chemical Society. Reproduced with permissions [30]. Copyright 2022, Wiley‐VCH. Reproduced with permissions [31]. Copyright 2024, American Chemical Society. Reproduced with permissions [10]. Copyright 2025, American Chemical Society.

In this review, we systematically examine the emerging role of p‐block metals in electrocatalysis for energy conversion (Figure 2). We first outline significant electrochemical reactions, focusing on the CO_2_, oxygen, and nitrogen‐based reactions. Other reactions are discussed that are of note or substantially challenging through conventional means. This review aims to provide the reader with a foundation of key application areas and current research directions (See Figure 3 for a summary of the major advances in *p*‐block metal‐based electrocatalysts for energy conversion over the last ten years [10, 23, 24, 25, 26, 27, 28, 29, 30, 31]). We discuss the unique properties of pure metallic forms and simple compounds of p‐block metals. Complex hybrid structures and composite materials are also explored, highlighting recent advances and potential future directions in the design of *p*‐block‐based electrocatalysts.

## 2 Metallic Forms, Crystals, Oxides, and Sulfides

Commonly, the p‐block metals and transition metals are combined, or the p‐block metals act as a supporting structure. Therefore, the literature on pure p‐block metal catalysis is sparse. However, to truly understand their catalytic abilities, a fundamental understanding of their properties is required. Focus should be on how they perform on their own or in simple materials/morphologies. Structurally, p‐block crystals tend to form layered or anisotropic lattices, which facilitate various bonding environments and interlayer interactions not found in d‐block systems [18, 32, 33, 34]. For instance, the interlayer compression of Bi crystals can tune orbital overlap and delocalization, directly impacting catalytic activity [13]. P‐block metals' structural adaptability perfectly fits into defect engineering, nanostructuring, and low‐dimensional applications. P‐block metal oxides and sulfides are also attractive for various applications. P‐block oxides are characterized by high oxophilicity, redox flexibility, and defect tolerance, which allows them to stabilize oxygen‐rich intermediates in ORR and CO_2_RR [35]. In contrast, sulfides exhibit narrower band gaps and higher electronic conductivity, facilitating charge transfer but often at the expense of long‐term stability [36]. Another hallmark is their ability to cycle between oxidation states (e.g., Bi^3^^+^/Bi^0^, Sn^4^^+^/Sn^2^^+^), which provides natural charge compensation during redox reactions and enhances durability under dynamic electrochemical conditions.

In summary, the unique electronic structure, structural flexibility, and adaptable oxidation states of p‐block metals can unveil new catalytic pathways and design principles. Instead of serving as lower‐cost substitutes for noble metals, p‐block systems provide an alternative conceptual framework for electrocatalysis. The field of p‐block metal electrocatalysts is growing, leveraging their distinctive chemistry to achieve high selectivity, tunability, and sustainability. Here, we will discuss recent works that focus on the performance of p‐block metals in various morphologies as discussed above.

### 2.1 Nitrogen Reduction Reaction (NRR)

Uniquely, pure p‐block metals have been recently studied for the NRR. The NRR is a cornerstone of modern agriculture as it is the primary source of NH_3_. Currently, the industrial ammonia process follows the Haber–Bosch method, where nitrogen and hydrogen react at high pressure and temperature with a Fe‐based catalyst in aqueous electrolytes [37]. These conditions cause high energy consumption and CO_2_ production. The electrocatalytic NRR mechanism is not fully understood, but there is a consensus on what occurs at the atomic scale (See Table 1) [38]. The generalized associative and dissociative mechanisms are both recognized and accepted within academia. The adsorption configuration determines the pathway the reaction will proceed. The dissociative pathway (Haber–Bosch process) is impossible under ambient conditions because of the breaking of the N triple bond [39, 40]. The N_2_ molecule exhibits pronounced thermodynamic stability, is resistant to weakening via electron transfer, and has a high HOMO‐LUMO gap [38]. As a result, no industry viable alternative to the 100‐year‐old method has emerged [41].

**TABLE 1:** Summary of NRR mechanisms and pathways.

**NRR**	Distal	* + N_2_ → NNH* → NNH_2_* → N* → NH* → NH_2_* → *
	Alternative‐distal mixed (I)	* + N_2_ → NNH* → NHNH* → NHNH_2_* → NH* → NH_2_* → *
	Distal‐alternative mixed (I)	* + N_2_ → NNH* → NNH_2_* → NHNH_2_* → NH* → NH_2_* → *
	Distal‐alternative mixed (II)	* + N_2_ → NNH* → NNH_2_* → NHNH_2_* → NH_2_NH_2_* → NH_2_* → *
	Alternative	* + N_2_ → NNH* → NHNH* → NHNH_2_* → NH_2_NH_2_*‐NH_2_* → *

* indicates the bare catalytic surface and any compound with * adjacent to it indicates its adsorption to the surface.[42]

**FIGURE 4:**
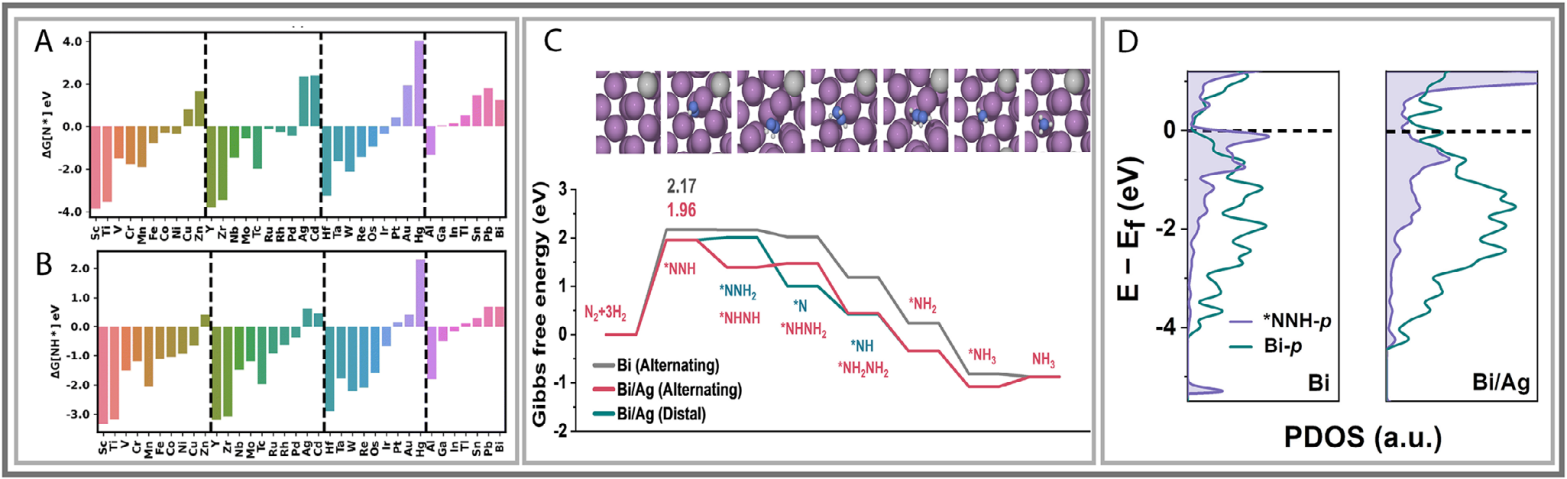
(A,B) Adsorption free energies (ΔG; eV) of NNR intermediates (i.e., N^*^ and NH^*^) on various metal nano clusters. Reproduced with permission [42]. Copyright 2025, Royal Society of Chemistry. (C) The pDOS of ^*^NNH‐p orbital and Bi‐p orbital on Bi0 and electron‐rich Bi^0^(Bi/Ag). (D) Gibbs free energy diagrams of ENRR for Bi^0^ and electron‐rich Bi^0^ (Bi/Ag). (C,D) Reproduced with permission [44]. Copyright 2023, National Academy of Sciences.

P‐block metals position themselves as an opportunity, offering unique advantages due to their tunable electronic properties and potential for facilitating N_2_ activation under milder electrochemical conditions. A recent publication by R.K. Raju (2025) had uncovered potential for p‐block catalysts [42]. In this work, pure p‐block and d‐block metal nanoclusters were compared for their NRR catalytic abilities. The free energy profiles suggest that the p‐block metals had the same favored mechanism as Pt, allowing for a direct comparison with PGMs. The p‐block metals observed negative changes in free energy, with instances that were lower than Pt. Like Pt, the N─N bond was broken on *p*‐block nanoclusters but lacked the required binding stability for protonating the N^*^ adsorbate. The *p*‐block nanoclusters overbind the NH^*^ and N^*^ species, producing a large overpotential (See Figure 4A,B). The p‐block metals acted as Lewis bases, accepting lone pairs of electrons and strengthening the adsorption [43]. Consequently, the strength of this adsorption was correlated to how full the p‐shell was and the element's electron affinity. The Al nanocluster exhibits only a partly filled 3p shell, which was insufficient for the π‐bond orbital between Al and N.

The selectivity of the pure *p*‐block metals was also mentioned to be poor by R.K. Raju's (2025) findings; however, other works have contradicted R.K. Raju's (2025) findings statements [14, 45]. In creating this review, it was challenging to develop a consensus as contradictory findings were common. There should be a significant push for solidifying our understanding of p‐block metal electrocatalysts. The metals in the boron group were known for the high NRR selectivity, specifically Al [43]. The strong initial activation of N_2_ and coupling of the Al and N orbitals stabilized the intermediate species before the competing reaction pathway. These factors influence how intermediates bind to the catalytic surface, which guides the formation of products [45]. Additionally, p‐block metals naturally limit the competing HER, which enables other nitrogen species to form. In these and similar studies, a key limitation of p‐block metals is their poor capacity to stabilize intermediate species. However, the stability of intermediates can be improved by manipulating morphology or electronic properties (which will be discussed in the next section).

The solution and electrolyte conditions have been known to impact the performance of many catalysts. The poor nitrogen solubility requires an electrolyte composition to control proton transfer and regulate preferable reaction pathways. However, it is often neglected when discussing electrocatalytic performance and should be studied more thoroughly. According to Y et al. (2019) [46], the NRR activity of Bi was significantly enhanced by potassium cations. The potassium ions act as charge carriers at the surface, improving charge transfer. The presence of the potassium bends and elongates the N─N bond, suggesting better N–N activation on bismuth surfaces in the presence of potassium cations. Other works have noted that potassium ions themselves polarize the N_2_ molecule, enabling faster activation and intermediate formation [47, 48]. However, Bi has demonstrated many desirable properties in many academic works.

When compared to Au, Bi significantly outperformed the noble metal in preventing HER, improving stability, and increasing activity. Bi holds significant potential on its own as a potential NRR catalyst and was often mentioned in the literature. A key strategy employed involves manipulating the oxidation state of the catalytic atom to maintain the ideal electronic states for adsorbate interactions. The metallic Bi^0^ species was ideal for catalytic NRR (See Figure 4C,D) [49, 50]. However, the Bi^3+^ species is prone to reduction, which changes the morphology and performance of the material. Therefore, supporting structures are required to maintain the current oxidation state for high performance. Additionally, through manipulating the surface potential of the Bi surface, the local electronic structure was tuned for enhanced performance.

Similar studies on Bi nanostructures have also demonstrated high catalytic performance (Faraday efficiency of ∼ 10.26% and potential of ∼−0.50 V vs. RHE) [49, 51]. In comparison, the local surface morphology was tuned to enhance NRR performance instead of the electrolyte environment. Nanosheets are an attractive and cheap substitute for PGM due to their scalable synthesis. The resulting nanostructure induced stronger interactions between the p orbitals of metal substrates and nitrogen adsorbates. The work recorded Bi 6p bands and the adsorbed N atoms 2p orbitals overlap both below and above the Fermi level, suggesting a strong interaction. Rong et al. (2019) work also found that some p‐block metals were better catalysts than others due to their varying p‐orbital configuration [51]. Al exhibits electrochemical behavior similar to early transition metals in several key electrochemical steps [42]. Al's performance was reminiscent of Fe or Ir, which both had low overpotentials but suffered from strong NH_2_ adsorption. Here, the sp^3^ hybridization between the NH_2_ 2p and Al 3s orbitals completes the π bond, which prevents the desorption of NH_2_ [52]. Therefore, finding the right electron configuration and physical properties is ideal for creating a catalyst for NRR.

### 2.2 Carbon Dioxide Reduction Reaction (CO_2_RR)

**FIGURE 5:**
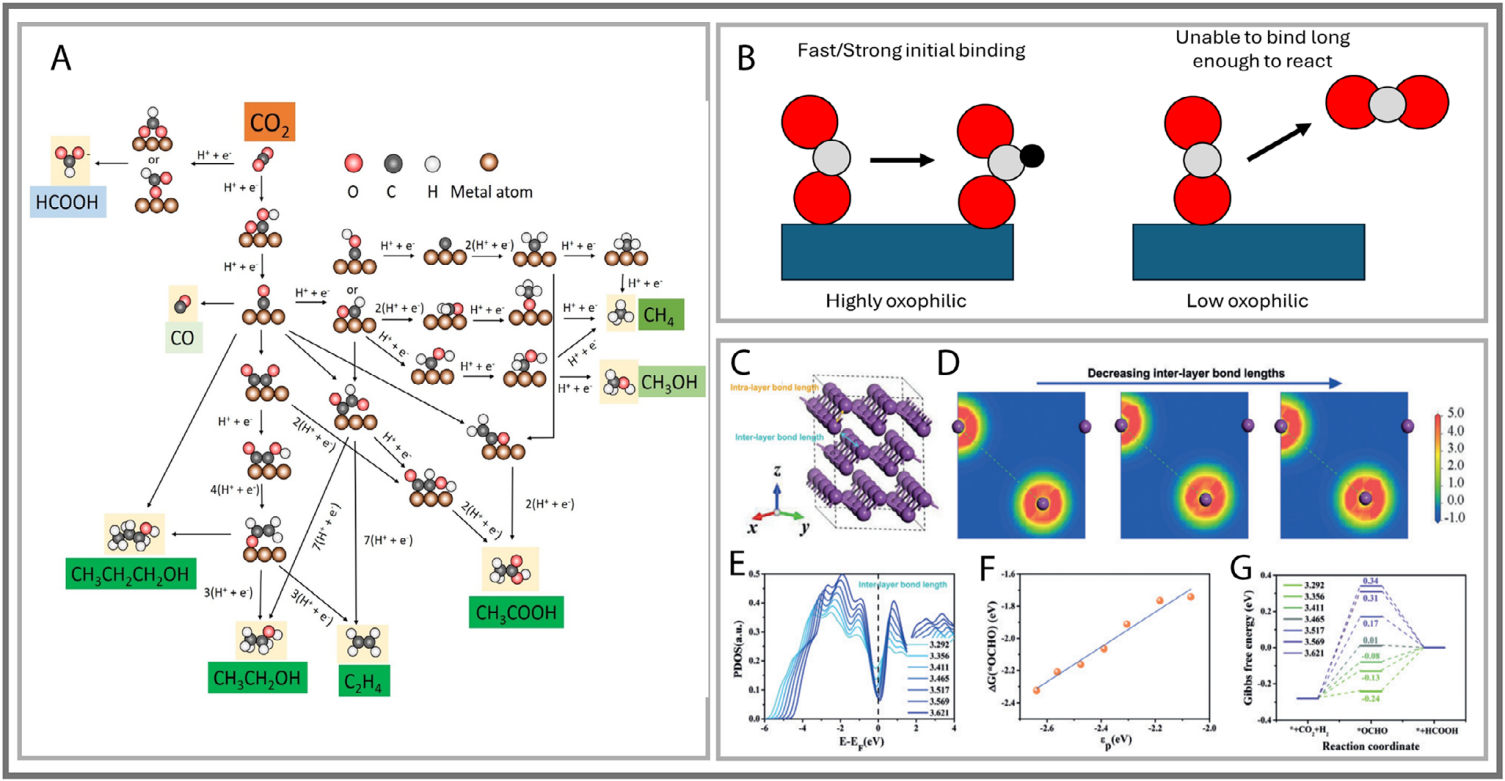
(A) Carbon dioxide reduction reaction scheme. Reproduced with permission [57]. Copyright 2022, Multidisciplinary Digital Publishing Institute. (B) Visualization of high oxophilic benefits in p‐block metal catalyst. The metal catalyst stronger binds to oxygen in adsorbates, which increases the breaking of stable molecules and reaction activation. (C) The crystal structure of bulk Bi in Vesta. (D) The change in electronic distribution between neighboring inter‐layer Bi atoms. (E) Partial density of States (PDOS) of the valance p‐orbital of differently spaced Bi atoms. (F) Discovered a relationship between p‐band center and adsorption free energy of ^*^OCHO for the formation of formate in CO_2_RR. (G) Calculated Gibbs Free Energy of formate formation in CO_2_RR at U = 0 V. (C—G) Reproduced with permission [13]. Copyright 2018, Wiley‐VCH.

The other reaction that has gained rapid attention for p‐block metals catalysts is the CO_2_RR. The CO_2_RR is a crucial reaction for CO_2_ removal and the production of fuels and chemicals, eliminating the need for fossil fuels [53, 54]. However, like many of the reactions discussed in this paper, molecular bonds are difficult to break without a catalyst [55, 56]. Additionally, the CO_2_ reaction is complicated with many proposed reaction pathways that result in multiple differing end products (see Figure 5A and Table 2) [57]. The key limitations of CO_2_RR catalysts are the large overpotential, large activation energy barriers, and poor selectivity. As there are many intermediate species in the reaction, the stability of these intermediate steps impacts those limitations and results in a spectrum of products [58, 59]. Au and Cu are excellent catalysts for the reaction as they are selective, resistant to degradation, and efficient [60, 61]. However, some p‐block metals have shown potential as an alternative with limited applications [62].

**TABLE 2:** Summary of CO_2_RR mechanism and pathways.

**CO_2_RR**	Distal C_1_	∗ + CO_2_ → COOH∗ → CO∗ → COH∗ → C∗ → CH∗ → CH_2_∗ → CH_3_∗ → ∗
	Alternating C_1_	∗ + CO_2_ → COOH∗ → CO∗ → CHO∗ → CH_2_O∗ → CH_3_O∗ → ∗
	Distal‐like C_2_^+^	2CO∗ → OCCO∗ → OCCOH∗ → CCO∗ → CCHx∗ → ∗
	Alternating C_2_^+^	2CO∗ → OCCO∗ → CHO–CHO∗ → CH_2_CHO∗ → CH_3_CH_2_O∗ → ∗
	CO	∗ + CO_2_ → COOH∗ → CO∗ → ∗
	Formate	∗ + CO_2_ → OCHO∗ → ∗

* indicates the bare catalytic surface and any compound with * adjacent to it indicates its adsorption to the surface [57].

Sn, In, Bi, and Pb, as representative p‐block metals, have recently been shown to catalyze CO_2_RR with excellent selectivity for producing formic acid and formate [45, 63, 64, 65, 66, 67, 68]. The selectivity of p‐block metals has been acknowledged for a long time [69], yet the reaction pathway has only been studied recently. The pure p‐block catalysts exhibit similar overpotentials (1.6–1.1 V vs. RHE), with the notable exception of Bi, which achieves a markedly reduced overpotential of 0.741 V [45]. The selectivity and activity toward the CO_2_RR were determined by the relative binding strengths toward the reaction intermediates (^*^OCHO, ^*^COOH, ^*^CO, and ^*^H) on the surface [53, 70]. The p‐block metals exhibit a high selectivity because of their weak interaction with H, which inhibits the competing HER. Additionally, the characteristic oxophilic nature of p‐block metals favors the formation of oxygen‐dominated intermediates like OCHO^*^ (over ^*^COOH), which is key for their selective production of formate and formic acid (see Figure 5B). The Bi catalyst preferred the highly efficient two‐electron transfer route, opening a commercial opportunity if further investigated [69, 71, 72, 73]. Again, Bi was a standout compared to other p‐block metals in this study, but incomparable to traditional Pt/C catalysts. Therefore, understanding the inherent nature of Bi and how to enhance its performance is essential if it is to be a competitive alternative.

Bi had been commonly paired with transition metals as a CO_2_RR catalytic support [13]. Yet, Bi had been a clear standout for electrocatalysis within the p‐block metals, but still needs to improve its catalytic performance to be viable. S. et al. (2018) noticed that the p‐orbitals of Bi can be delocalized by varying the interlayer distance [13]. The adsorption energy of the intermediate ^*^OCHO increases with the shortening of the interlayer Bi─Bi bond length (See Figure 5C–G). The decrease in adsorption energy reduces the activation barrier of the first protonation of CO_2_, and correspondingly enhances the performance and selectivity toward formate. Interestingly, when the inter‐layer Bi−Bi bond length decreases to 3.36 Å, the overpotential of ^*^OCHO is predicted to be near‐zero (0.01 eV). The resulting Faradaic efficiency was 95% at −0.86 to −1.36 V vs. RHE with high selectivity. These findings suggest that optimizing the degree of p‐orbital delocalization via interlayer compression provides a powerful design strategy for enhancing formate selectivity in CO_2_RR. More broadly, this highlights how orbital‐level engineering beyond conventional d‐band concepts can be exploited in p‐block metals to break scaling relations, lower overpotentials, and open new pathways for selective electrocatalysis [74].

### 2.3 Oxides and Sulfides

**FIGURE 6:**
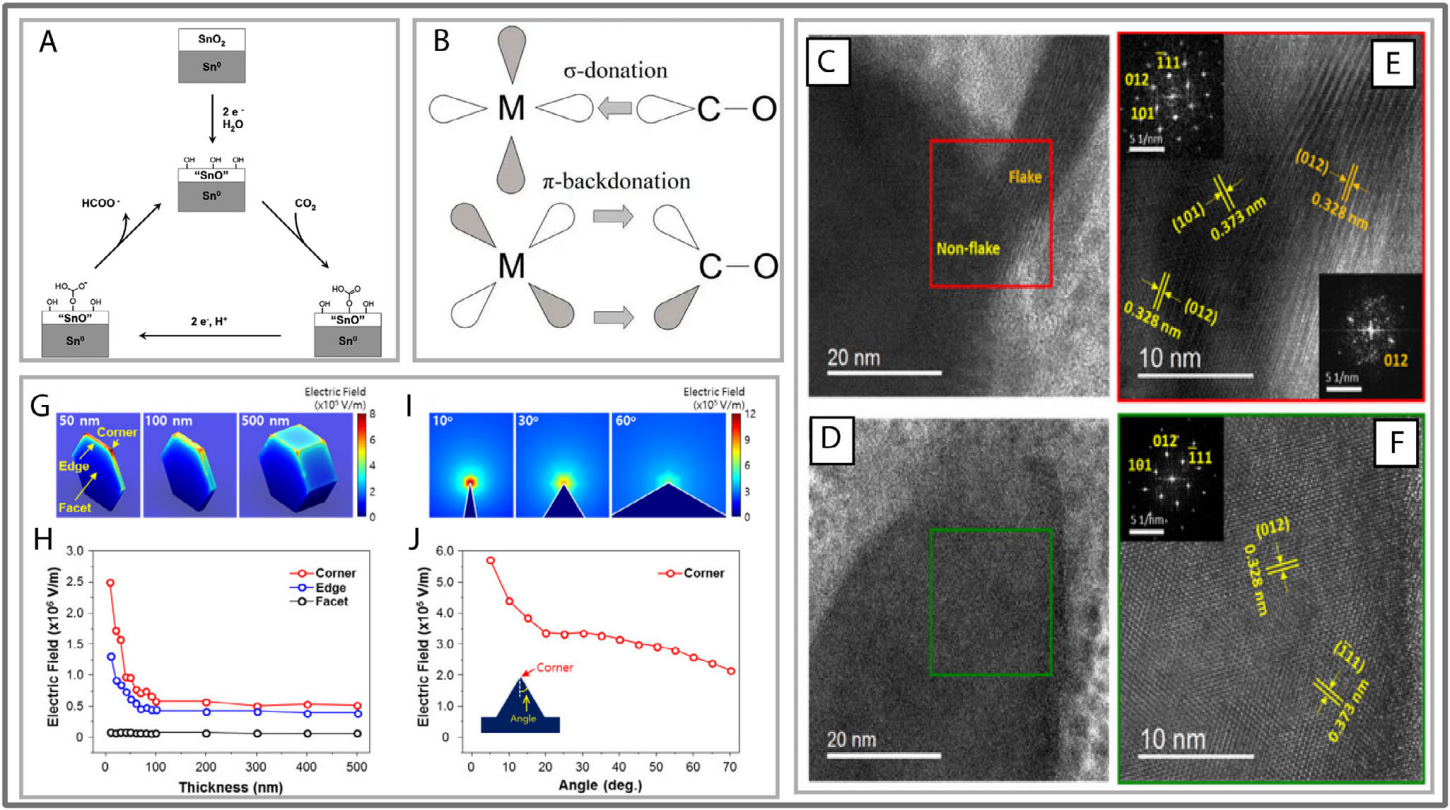
(A) Schematic diagram of the suppositional mechanism of CO_2_ reduction to formate on the Sn/SnO_x_ electrode. “SnO” on Sn^0^ refers to a Sn^II^ oxyhydroxide species. Reproduced with permission [82]. Copyright 2015, American Chemical Society. (B) General example of π back donation for transition metal adsorption. Reproduced with permission [83]. Copyright 2023, Elsevier Ltd. (C,D) Characterizations of Bi nanostructures. High resolution TEM (HR‐TEM) images of (C) Bi nanoflake in PC‐6c and (D) Bi nanodendrite in DC‐120s. Corresponding (E) red and (F) green square images showing crystalline planes of (012), (101), and (111) of Bi. (G–J) Simulated electric field distribution in (G) 3D and (H) 2D Bi nanostructures; a strong electric field is formed as the shape is thinner and sharper. Electric field intensity plotted as a function of (I) thickness and (J) corner angle of Bi nanostructure. (C–J) Reproduced with permission [20]. Copyright 2017, Elsevier Ltd.

P‐block metal oxides and sulfides have gained growing interest as catalysts, especially due to their potential as substitutes for noble metals. These materials show promise for being chemically stable, redox flexible, and defect‐tolerant [75]. The surfaces of metal oxides and sulfides can exhibit acidic, basic, or amphoteric character depending on their composition and oxidation state, enabling reaction‐specific tuning of catalytic activity [76]. This tunability is one of their most underappreciated advantages compared to transition metal oxides, which often show narrower redox flexibility. For example, the metal oxide surface terminates in the O^−2^ ion, whose large size promotes a defect‐rich environment. Metals such as Sn and In depend on their corresponding metal oxides to enhance catalytic performance and selectivity. For Sn, the SnO_2_ and SnO forms regulate the electron flow and ensure the correct oxidation state is present for high Faradaic efficiency (see Figure 6A) [45, 77, 78]. Meanwhile, In utilizes its oxide form to produce alkali hydroxides that better dissolve CO_2_ into the water, but also allow for the selective production of CO_3_ [79, 80, 81]. These findings suggest that oxides are not merely passive surface layers but essential co‐catalysts, a perspective that is often overlooked in the current literature.

The electronic structure of p‐block oxides also enables back‐donation effects traditionally reserved for transition metals. In conventional catalysis, d‐orbitals back‐donate to ligand π^*^ orbitals. However, Bi_4_O_5_I_2_ mimics this behavior through its p‐orbitals, demonstrated by Lv et al. (2020) [22, 84]. Therefore, this knowledge expands the theoretical toolkit for catalytic design, as not only d‐block metals can exploit back‐donation (see Figure 6B). Furthermore, the parallel with Bi interlayer compression underscores a broader theme: structural flexibility in p‐block systems can create electronic environments usually associated with transition metals [13]. Despite these exciting results, the reported catalytic metrics remain modest (e.g., 20.44 µg h^−^^1^ mg^−^^1^ NH_3_ yield, 3.24% Faradaic efficiency under acidic conditions) [22, 84]. This highlights a critical gap: while p‐block oxides demonstrate fascinating electronic behavior, their practical catalytic performance lags far behind expectations. Future work must shift from mechanistic proof‐of‐concept studies to strategies that stabilize these effects under operating conditions and integrate them into scalable architectures. Among recent advances, the work of Ye et al. (2025) stands out as one of the most impressive demonstrations of a p‐block oxide catalyst for CO_2_‐to‐formate conversion [85]. By implementing a Turing‐style topology, they were able to finely tune the oxophilicity of Sb_0_._1_Sn_0_._9_O_2_. Their design exploited ionophilicity to reorient interfacial water molecules, selectively stabilizing ^*^OCHO intermediates over competing HER pathways. This was elegantly captured in a volcano‐type relationship: weak oxophilicity suppressed ^*^OCHO adsorption, while excessive oxophilicity favored hydrogen evolution due to increased proton donation from structured water networks. The optimized Turing‐structured catalyst achieved a remarkable Faradaic efficiency of 92% at 1000 mA cm^−2^ and remained stable at 500 mA cm^−2^ for over 200 h in a membrane electrode assembly. These results highlight a key point often overlooked in the literature: oxophilicity is not a static property but can be topologically engineered to optimize both adsorption and interfacial water dynamics. This reframes the design rules for CO_2_RR catalysts, suggesting that surface topology is just as critical as composition.

Oxygen can also contribute synergistically when both O and S are present, leading to mixed‐anion frameworks with enhanced redox flexibility [86]. The multiple valence states of many p‐block metals (e.g., Bi^3^^+^/Bi^0^, Sn^4^^+^/Sn^2^^+^) allow for charge compensation during cycling, improving stability and ion tolerance [87, 88]. However, it's worth noting the distinctions between oxides and sulfides: oxides tend to have higher ionic bonding energies and stability, while sulfides typically have narrower band gaps and higher conductivity [35].

Despite these promising features, p‐block metal sulfides remain underexplored relative to their oxide counterparts. From recent studies, Sn, Bi, and Pd are the most common sulfides for various reactions and morphologies [88, 89, 90]. Bi_2_S_3_ was recently used for CO_2_RR and demonstrated excellent electron transport properties [91, 92]. Wang, L. (2023) investigated nanoflower and nanofiber bunch morphologies, which achieved a high Faradaic efficiency of 96% at 0.75 V vs. RHE [91]. Other studies identified that higher performance metrics can be achieved through similar nanomaterial designs (Faraday efficiency of ∼80% at −0.4 V and ∼100% at −0.6 V) [20, 93]. For example, nanoflakes exhibit intersecting planes, which increase surface area and enable the highly active steps and edges to be accessible to the large CO_2_ molecules (See Figure 6C–F). Additionally, Kim, S, et al. (2017) paper demonstrated a relationship between the sharpness of the nanoflake's edges and the catalytic efficiency [30]. At sharp peak ranges, an electrostatic field was observed between the Bi_2_S_3_ and the surrounding electrolytes (see Figure 6G–J). The electric field repelled competing hydrogen atoms and improved the charge transport. However, the durability of these rough materials was limited. Often, these nanoflakes maintain their high performance for 8 h under ideal conditions before degradation occurs [20, 93]. Regardless, greater emphasis should be placed on surface morphology, as a detailed understanding of these structures and strategies to prolong their stability is still lacking.

The p‐block metal catalyst represents a compelling class of emerging electrocatalysts for a wide range of technologies. Even under simple morphologies and compositions, they exhibit desirable catalytic properties. However, challenges remain, including achieving competitive current densities and durability. Recent advancements have focused on electronic tuning via morphological control, demonstrating both affordability and effectiveness. Bi‐based catalysts have shown the most promise for NRR and CO_2_RR; however, they are still not competitive. PGMs exhibit lower overpotentials, faster charge transfer rates, and higher Faradaic efficiencies compared to p‐block sulfides, oxides, or their pure metallic forms. The limited knowledge of heavier p‐block metal oxides and sulfides, combined with their potent toxicity, makes commercial use difficult. There are several other limitations, including durability under electrolyte environments, poor conductivity, inferior current densities, and lower oxidation states. As research progresses, one popular strategy can further optimize p‐block metal catalysts. Single‐atom catalysis (SAC) presents the potential to maximize atomic utilization, enable precise control of active sites, and further tailor electronic environments. The following section will explore how incorporating p‐block elements into single‐atom frameworks addresses current limitations and unlocks new possibilities in electrocatalytic applications.

## 3 Single‐Atom Catalysts (SAC)

**FIGURE 7:**
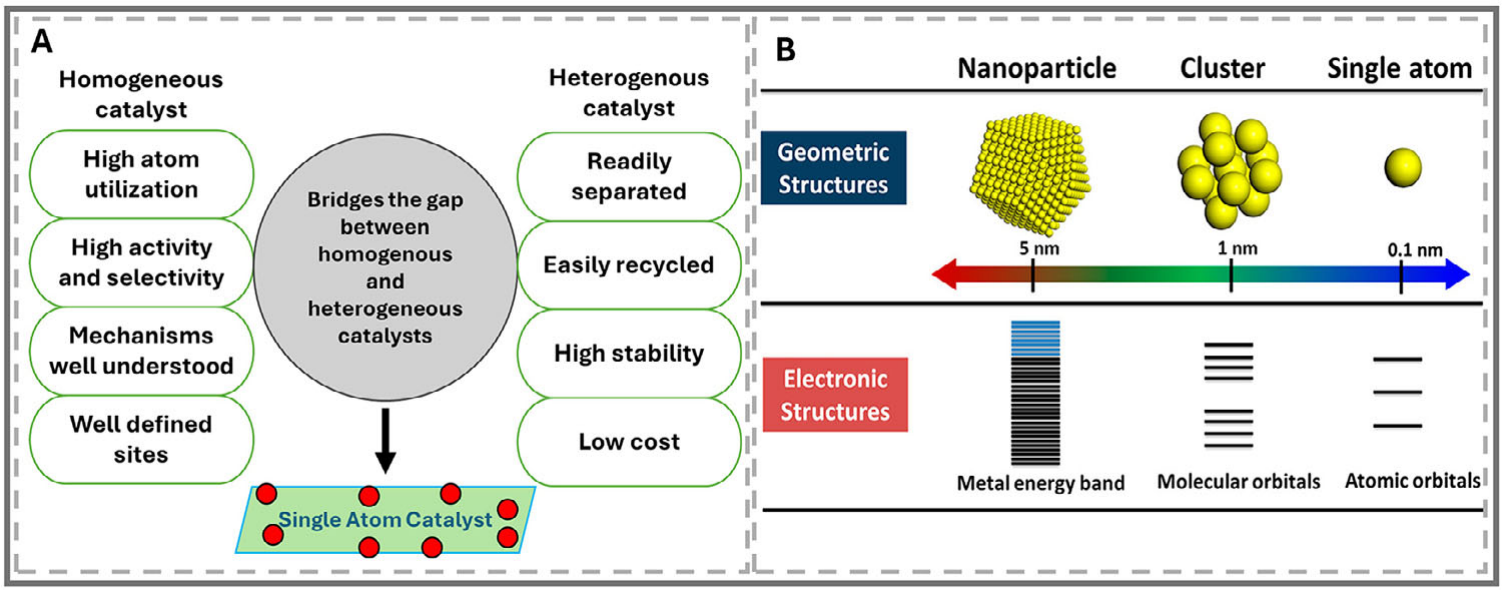
(A) SACs combine the advantageous properties of both homogeneous and heterogeneous catalysts. Reproduced with permission [97]. Copyright 2024, Elsevier Inc. (B) Geometric and electronic structures of single atom, clusters, and nanoparticles. Reproduced with permission [98]. Copyright 2018, American Chemical Society.

Precise control over the hybridization and electronic properties of a single atom can yield a highly selective and active catalyst [94, 95]. SACs, which are isolated atoms anchored to supports, represent a versatile platform for advancing electrocatalysis (See Figure 7A) [94, 95]. While early SAC research was primarily concerned with transition metals and gave limited attention to local coordination [96], recent findings show that modifying the surrounding atomic environment is equally essential. The specific atom and coordination environment determine the electronic structure of SACs. Strong binding through ionic or covalent interactions is required to firmly anchor single atoms in place, which induces significant charge transfer and prevents agglomeration [94]. The delocalization effect broadens adsorbate bands and enables the optimization of adsorption energies for targeted adsorbates (See Figure 7B). However, this very strength introduces vulnerability: the unique electron configurations that give SACs high selectivity also leave them prone to poisoning and takeover by competing reactions [94]. This trade‐off is at the heart of the SAC challenge: stability vs. activity. For p‐block systems, which inherently favor selective oxygenated intermediates, the question is whether they can balance these competing demands better than d‐block SACs.

### 3.1 Nitrogen Oxide Reduction (NOR)

**FIGURE 8:**
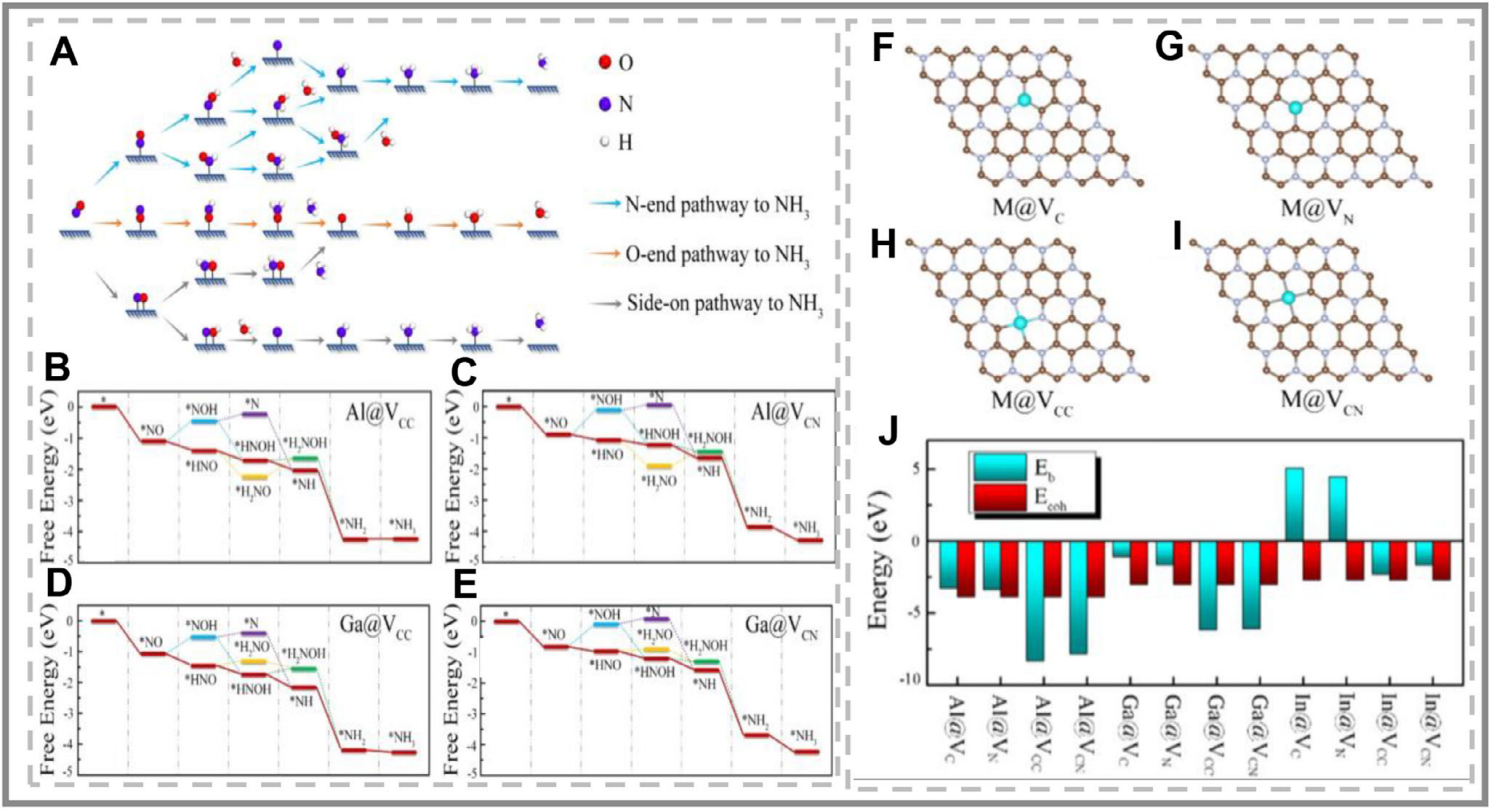
(A) Schematic of the reduction of NO to NH_3_. (B–E) Computed Gibbs Free energy diagrams of NO reduction on Al@V_CC_, (C) Al@V_CN_, (D) Ga@V_CC_, and (E) Ga@V_CN_ systems, respectively. (F–I) Top down view of main‐group p‐block metals (M = Al, Ga, and In)‐doped C_3_N monolayers. The brown, powder blue, and cyan balls stand for carbon, nitrogen, and main‐group metal atoms, respectively. (J) Binding energies of M@VC, M@VN, M@VCC, and M@VCN systems. The corresponding bulk cohesive energies (*E_coh_*) are also given for comparison. All reproduced with permission [101]. Copyright 2025, American Chemical Society.

A notable example is the conversion of nitrous oxide (NO) to ammonia, a process that is both environmentally critical and mechanistically challenging [99, 100]. In this reaction, the initial adsorption of NO is the primary challenge for catalysts. Interestingly, a recent study focused on p‐block SACs that demonstrated particularly favorable adsorption energies for this molecule (see Figure 8A–E). A computational study by Y et al. (2025) focused on SAC p‐block metal (Al, Ga, and In) doped onto C_3_N monolayers for NO reduction [101]. Al‐ and Ga‐based SACs have proven to be ideal systems for NO adsorption, exhibiting a preference for binding through the nitrogen atom of the NO molecule. The resulting chemisorbed bond produced a large charge transfer, facilitating the cleavage of the N─O bond. The strong interaction resulted from the adsorbing NO π‐antibonding orbitals strongly overlapping with the p‐orbitals of Al/Ga atoms around the downshifted Fermi level. This back donation was effective for NRR SAC as described by J. Wang (2021) [102]. The significant hybridization of occupied NO‐σ orbitals and p‐orbitals of the Al/Ga atom led to the formation of an occupied p‐σ bond below the Fermi level. Given its effectiveness, this backdonation mechanism should be prioritized in the design of other p‐block metal catalysts.

In and Sb SACs on MoO_3_ substrates also proved effective NORR catalysts, with isolated Sb sites showing enhanced NO binding while suppressing HER [103]. By isolating a localized Sb site on the SAC, the Sb atom exhibited few electronic states crossing its Fermi level, resulting in increased NO adsorption. This electronic configuration also weakened the water‐Sb interaction, preventing the competing HER. Vacancies and defects added another dimension: Al‐ and Ga‐doped C_3_N with engineered vacancies exhibited spontaneous reaction pathways and improved selectivity (See Figure 8F–J) [101]. Noticeably, vacancies are an effective way to initialize a challenging activation energy barrier and should be exploited in future SAC designs. This emphasized the importance of further research into defect engineering for SACs. Yet, the long‐term stability was uncertain and widely considered the main obstacle for practical applications [104]. Furthermore, the potential restructuring of the single atom under changing conditions led to questions about where the ‘true’ active site resided. Was it genuinely a single atom, or does it evolve throughout its lifetime as it undergoes structural change?

### 3.2 Oxygen Reduction Reaction (ORR)

**FIGURE 9:**
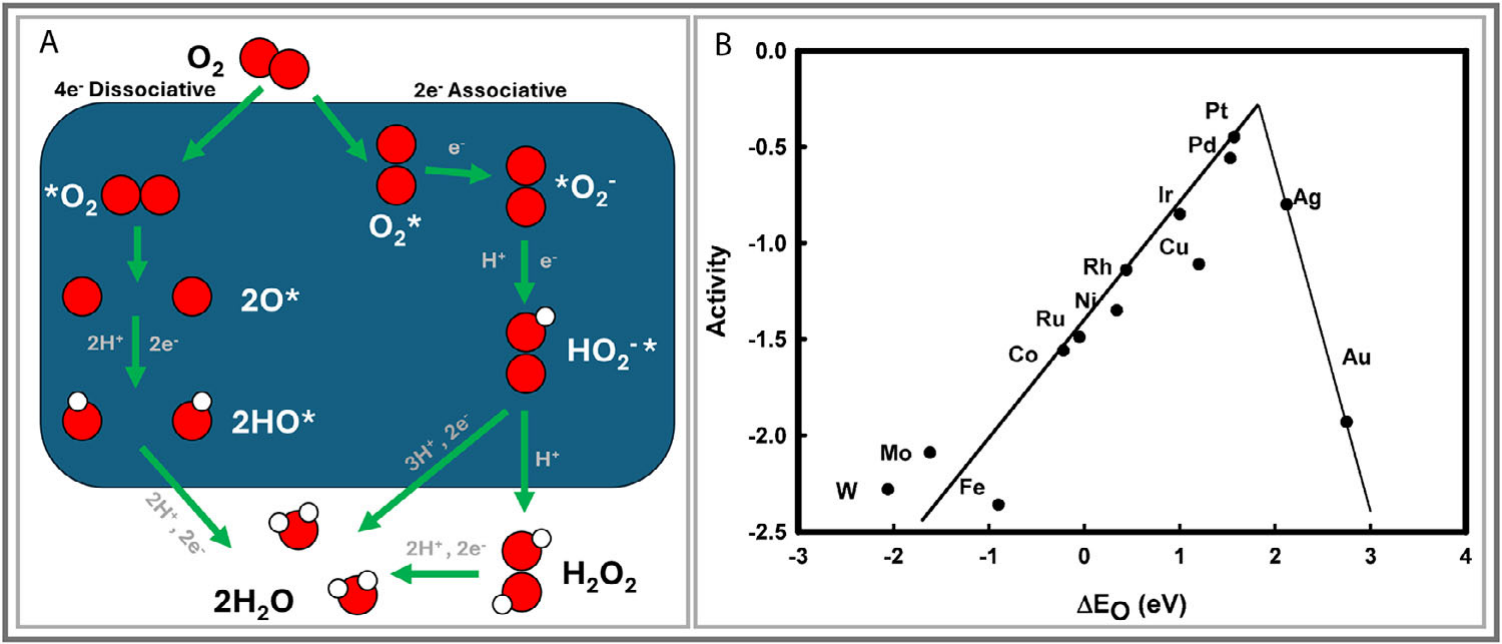
(A) Associative and dissociative oxygen reduction reaction. Reproduced with permission [107]. Copyright 2015, Multidisciplinary Digital Publishing Institute. (B) Trends in oxygen reduction activity plotted as a function of the oxygen binding energy. Reproduced with permission [108]. Copyright 2004, American Chemical Society.

The oxygen reduction reaction (ORR) remains one of the most formidable bottlenecks for electrochemical energy technologies, particularly in fuel cells. The ORR generally follows two pathways, the associative and dissociative mechanisms (See Figure 9A) [105, 106, 107]. For further clarification, Table 3 provides a detailed summary of the possible electrocatalytic ORR pathways [106]. Platinum group metals (PGMs) remain the benchmark catalysts, with Pt delivering exceptional activity owing to its ideally positioned d‐band center and favorable oxygen adsorption [105]. However, reliance on PGMs is fundamentally unsustainable given their scarcity and cost. The challenge for alternatives lies not just in replacing Pt but in overcoming ORR's inherent problems: sluggish kinetics, large activation barriers, ambiguous mechanistic pathways, and parasitic side reactions [106].

**TABLE 3:** Summary of ORR mechanism and pathways.

**ORR**	Dissociative	∗ + O_2_ → 2O∗ → OH∗ → ∗
	Associative	∗ + O_2_ → O_2_∗ → OOH∗ → O∗ + OH∗ → OH∗ → ∗
	Associative Alternating	∗ + O_2_ → O_2_∗ → OOH∗ → HOOH∗ → OH∗ → ∗
	2e^−^ Peroxide	∗ + O_2_ → OOH∗ → ∗

* indicates the bare catalytic surface and any compound with * adjacent to it indicates its adsorption to the surface [53].

P‐block metals provide a fundamentally different electronic platform to address these barriers. Unlike transition metals, which follow predictable d‐band scaling relationships, p‐block catalysts can break free of these constraints [108, 109]. Luo, F. et al. (2020) demonstrated the use of a Sn‐based single‐atom catalyst for acidic ORR [27]. Atomically dispersed Sn cations anchored in N‐doped carbon matrices showed four‐electron selectivity superior to traditional Fe–N–C or Co–N–C catalysts [27]. Crucially, the SnN_x_ moieties disrupted the linear scaling relationship between oxygen intermediate binding strength and catalytic activity. This approach bypassed the ‘volcano’ limitation that limits d‐block metals, representing a conceptual breakthrough (see Figure 9B) [108]. It demonstrated that p‐block SACs did not merely replicate transition metals but followed distinct coordination‐driven rules.

Furthermore, the weaker coordination of the Sn ion during O adsorption created a defect in the support material via long‐range vdW forces, which improved the free energy of formation. Thus, the reaction is only limited by the elementary step O^*^ ➔ OH^*^. As a result, p‐block metal SACs present a promising opportunity for catalyzing the oxygen reduction reaction (ORR). However, this opportunity comes with caveats. SnNC was shown to be highly vulnerable to impurities such as CO_2_, a ubiquitous component in real fuel‐cell environments [110]. This weakness underscores a broader issue: p‐block SACs often excel under controlled conditions but face durability and selectivity challenges in complex electrolytes. Without strategies to stabilize their coordination environment, they risk remaining laboratory curiosities.

**FIGURE 10:**
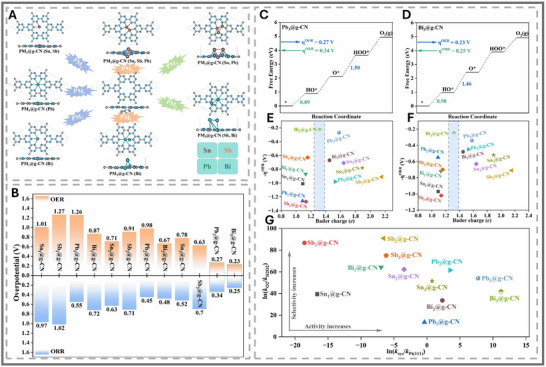
(A) Top and side views of the optimized configurations of PM1@g‐CN, PM2@g‐CN, and PM3@g‐CN electrocatalysts. (B) Summarized the OER and ORR overpotential on all the designed PMx@g‐CN catalysts. Calculated free energy diagrams of the OER and ORR on the designed (C) Pb3@g‐CN and (D) Bi3@g‐CN at zero potential. The blue and green values are the potential‐determining step values for the equilibria of the OER and ORR. (E) Correlation between ‐ηOER and the Bader charge of PM active centers of the designed catalysts. (F) Correlation between ‐ηORR and the Bader charge of PM active centers of the designed catalysts. (G) Variations of the selectivity vs. activity of the designed catalysts. All reproduced with permission [112]. Copyright 2024, American Chemical Society.

While utilizing a single atom as an active site offers significant advantages, recent research has increasingly focused on double‐ and triple‐atom catalysis (DAC and TAC) (See Figure 10A). The DACs and TACs not only inherit most of the advantages of SACs but also possess a higher loading of metal atoms. However, the most desirable outcome is the synergistic interaction between metal atoms, which enhances intrinsic catalytic performance [111]. Yana et al. (2024) focused on studying graphite‐like carbon nitride monolayer with anchored monomer, dimer, and trimer (denoted as PM*x*@g‐CN; PM = Sn, Sb, Pb, and Bi; *x* = 1–3) as effective non‐noble bi‐functional catalysts for both oxygen reduction and evolution reactions (see Figure 10B) [112]. Here, they identified that Bi_3_@g‐CN and Pb_3_@g‐CN had the lowest overpotentials for both reactions compared to any of the other p‐block metal configurations (see Figure 10C,D). The adsorption of HO^*^, O^*^, and HOO^*^ intermediates on Bi_3_@G‐CN induces hybridization with the Bi atoms. The partially occupied p electronic states of Bi atoms mimic d electronic states in a similar effect to back donation. Therefore, it induces an ideal localized charge environment that promotes ORR and OER (see Figure 10E–G). This synergy results in overpotentials lower than those of IrO_2_ and Pt(111), the current commercial standards [53, 113]. Therefore, p‐block multi‐atom sites could constitute a fundamentally new class of precise catalysts, rather than merely serving as substitutes for PGMs.

### 3.3 Applications in CO_2_RR

**FIGURE 11:**
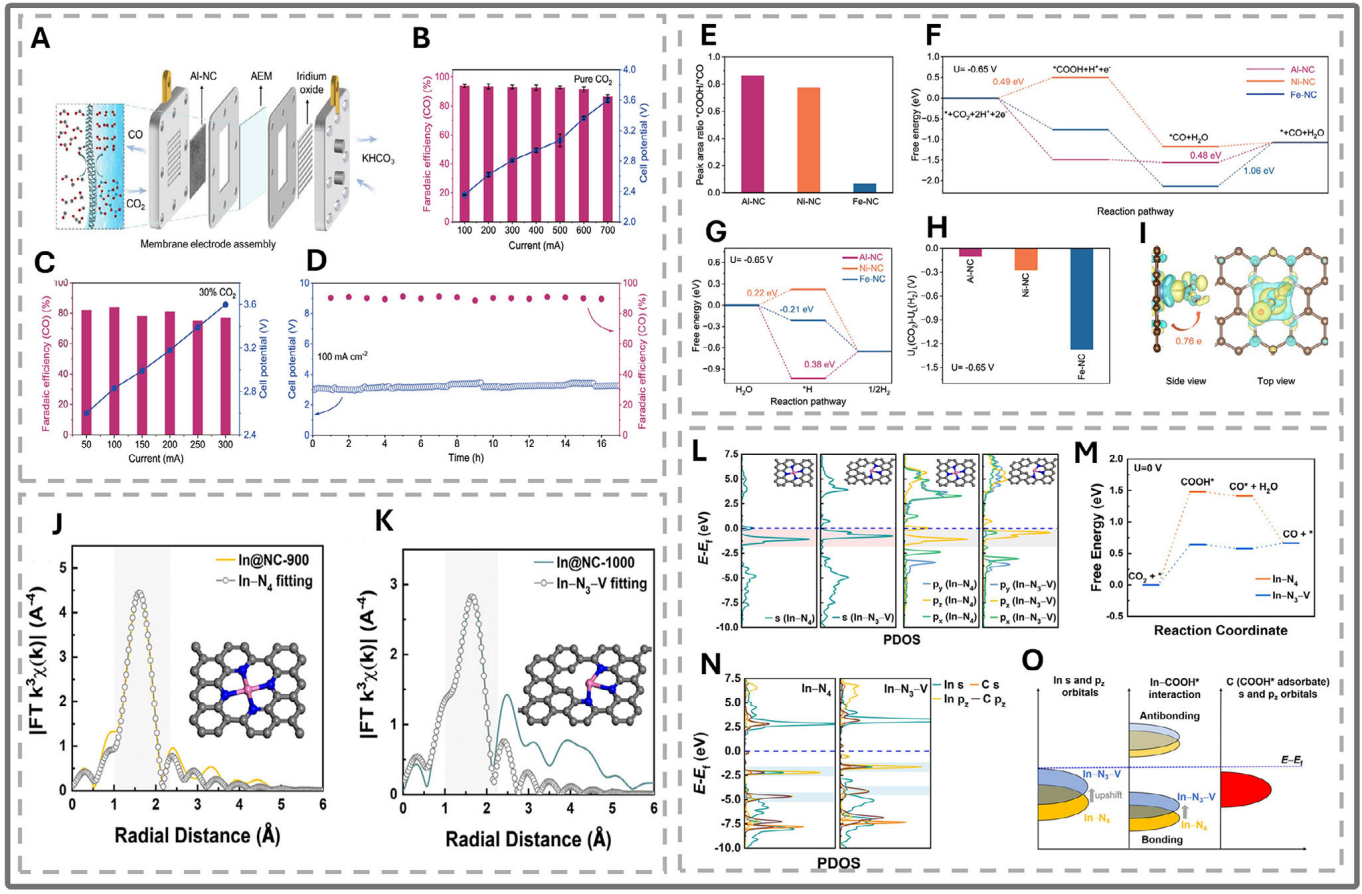
(A) Schematic illustration of the membrane electrode assembly (MEA) device. (B,C) Potentials and FECO at different current densities of Al–NC in MEA under varying concentrations of CO_2_ as the feedstock pure CO_2_ (B) and 30% CO2 (C). (D) Long‐term stability of Al–NC in MEA by using pure CO_2_ as feedstock. (A–I) Reproduced with permission [114]. Copyright 2024, American Chemical Society. (E) Peak area ratio of ^*^COOH/^*^CO at −0.7 V vs. RHE. (F,G) Calculated free energy diagram for CO2 to CO (F) and H2O to H2 (G) conversion for Al–NC, Ni–NC, and Fe–NC at U = −0.65 V. (H) UL(CO_2_) – UL(H_2_) values of Al–NC, Ni–NC, and Fe–NC at U = −0.65 V. (I) Electron density difference plot of the *COOH intermediate adsorption on Al–N_4_ and Bader charge analysis. (J) EXAFS r‐space fitting of In@NC‐900. (K) EXAFS r‐space fitting of In@NC‐1000. (L) Projected electron density of states (PDOS) of In s and p orbitals in In–N4 and In–N3–V models. (M) Calculated free energy diagram for CO2‐to‐CO conversion using In–N_4_ and In–N_3_–V models. (N) PDOS of In (s and pz orbitals) in In–N4 and In–N3–V interacting with C (s and pz orbitals) in COOH^*^. (O) Cartoon schematic PDOS illustration of the differential In orbital energy and In–COOH^*^ bond formation on In–N4 and In–N3–V models. (J–O) Reproduced with permission [116]. Copyright 2022, American Chemical Society.

Al SACs are highly active and readily reduce CO_2_. Ma, Z et al. (2024) recently demonstrated that Al on a nitrogen‐doped carbon structure outperforms comparable structures with Ni or Fe in a membrane electrode (see Figure 11A) [114]. This Al SAC maintained a 95% CO selectivity under a 100mA cm^−2^, with minimal degradation. When the current was raised to 700 mA, the cell voltage climbed to 3.6 V, accompanied by a CO selectivity of 86% (see Figure 11B,C). At such voltages, H_2_O_2_ generation via competing reactions is a liability for the membrane's integrity [115]. However, Al SAC offers good stability in a membrane electrode assembly (see Figure 11D). After a slight decrease in selectivity to 94%, the current density of 400 mA was sustained for 16 h of CO_2_RR electrolysis in a membrane electrode cell [114].

Density functional theory (DFT) demonstrated that the competing hydrogen adsorption was a large energy cost, while the energy barrier for COOH^*^ formation was small (See Figure 11E–I). The presence of substantial charge migration between the graphene and Al atoms contributed to the high current densities. In Fe‐NC, CO and H overpower other adsorbates, leading to gradual degradation of the catalyst. The durability of SACs was also contingent upon the stability of the metal atom. When adsorbates bind to the catalytic atom, the surface energy changes, leading to migration of the single atom onto the support material or membrane [117]. As a result, they are not currently viable under extreme conditions like high temperatures or pressures [118]. Furthermore, a fundamental understanding of the thermal stability of SACs in regeneration conditions is needed, which is helpful for the regeneration strategy design and optimization. In the future, a great effort should focus not only on the effectiveness of the catalyst but also on the lifetime of the catalyst and electrode to be competitive and an effective technology [117].

As an environmentally friendly *p*‐block metal, indium (In) has attracted recent interest for CO_2_RR applications. Previous studies showed that In‐N_4_ selectively catalyzed the conversion of CO_2_ to CO with 97% efficiency by lowering the CO_2_ energy barrier [119]. However, this reaction required a solution of acetonitrile, which is highly toxic and expensive [120]. S. et al. (2022) proposed two alternative SAC that remove the toxic electrolyte [116]. In‐N_3_‐V is a nitrogen‐doped graphene sheet with a single In atom as the active site (see Figure 11J,K). The In atom is adjacent to a vacancy site, which was shown to promote catalytic activity and selectivity. The reported potential was 0.57 V vs. RHE, with a current density of 2.3 mA cm^−2^ and a Faradaic efficiency of 95.1%, exceeding or being on par with transition metal SACs [121, 122, 123]. The key electronic mechanism for this improved performance is the shifting of In's S and Pz orbitals (see Figure 11L,M). As they move closer to the Fermi level, the ^*^COOH intermediate binding energy strengthens, which then allows CO production (see Figure 11N). The HER reaction was outcompeted by ^*^COOH as it has a larger activation energy. In‐based catalysts are known to favor formate production, whereas In‐N_3_‐V encounters a substantial energy hurdle to generate this product [81, 124, 125]. As mentioned above, the Pz orbital is critical for ^*^COOH binding. The difference in molecular orbital shapes between HCOO^*^ and ^*^COOH accounts for the preference for CO, as demonstrated in recent research (see Figure 11O) [75]. Therefore, the structure of the SAC is an important indicator of catalytic performance, and the p orbitals of p‐block metals hold potential toward untapped catalytic abilities.

**FIGURE 12:**
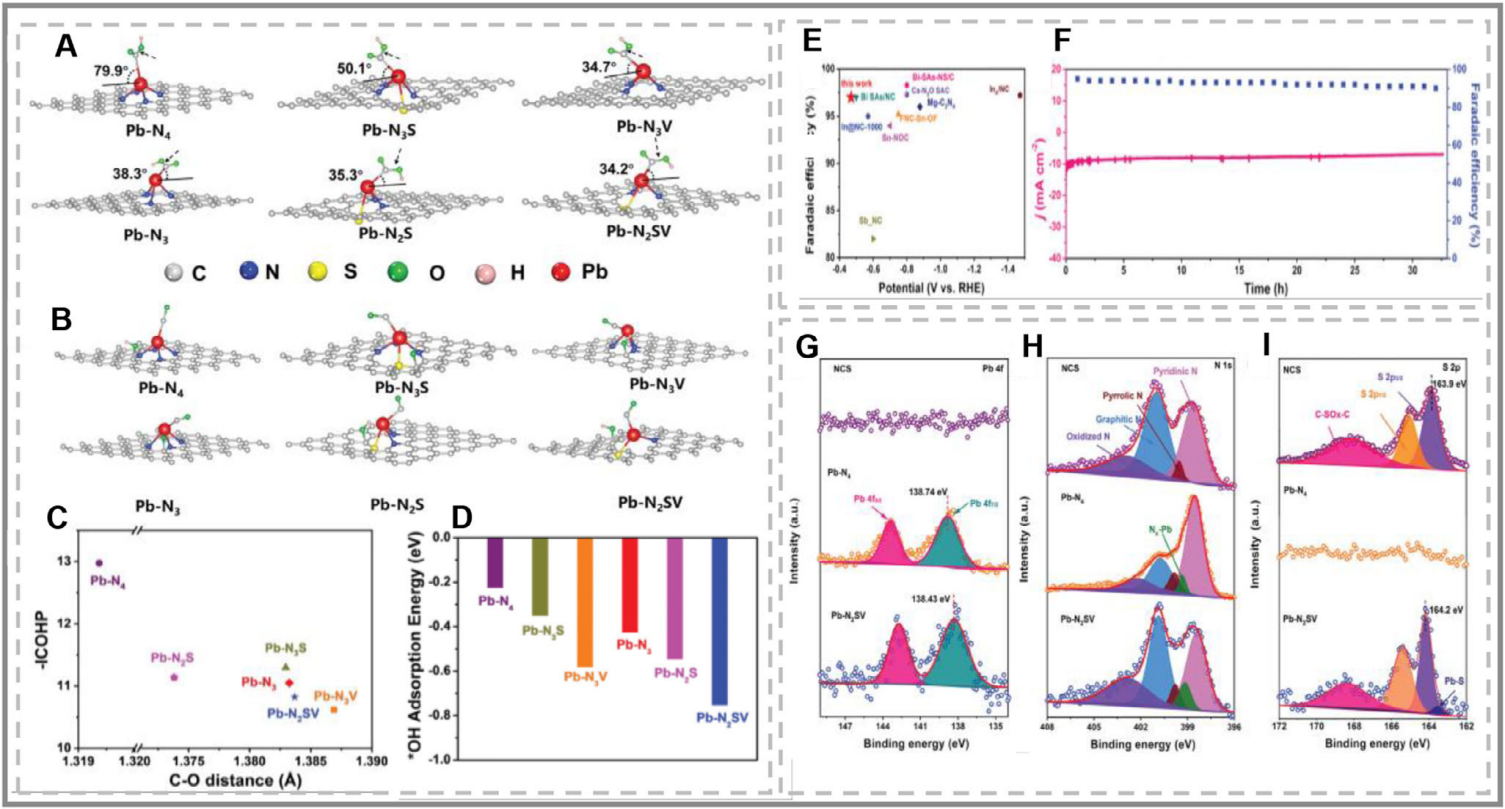
(A) Pb SACs models interacting with ^*^COOH. (B) Pb SACs models interacting with ^*^CO and ^*^OH. (C) ‐ICOHP values for the interaction between C and OH in ^*^COOH. (D) adsorption energies of ^*^OH over Pb SACs models interacting with ^*^CO. (E) Comparison of the highest FE_CO_ for Pb‐N_2_SV at −0.47 V and reported main‐group metals SACs. (F) The long‐time durability test of Pb‐N_2_SV at the potential of −0.57 V in CO_2_‐saturated 0.5 m KHCO_3_. XPS analysis of (G) Pb 4f, (H) N 1s (I) S 2p for NCS, Pb‐N_4_ and Pb‐N_2_SV. All reproduced with permission [126]. Copyright 2023, Wiley‐VCH.

In 2023, Zhou S‐H et al. identified a novel Pb‐based SAC with high asymmetry, demonstrating exceptional CO_2_RR performance [126]. A Pb atom is coordinated between two nitrogen atoms, one sulfur atom, and an adjacent vacancy (see Figure 12A,B). The deliberate break in coordination symmetry finely tuned the electronic structure of the Pb active site (see Figure 12C,D). The resulting performance struck a near‐optimal balance between the adsorption of COOH and the desorption of CO. The Pb‐NSV catalyst exhibited a Faradaic efficiency of 97.3% for CO at an overpotential of ‐0.47 V, with an operational lifetime over 33 h (see Figure 12E,F). This catalyst already outperforms other conventional SACs [127]. However, introducing an S dopant to the structure resulted in stronger binding between the Pb p orbitals and C p orbitals (in the intermediate ^*^COOH molecule) (see Figure 12G–I). The presence of the S dopant promoted electron accumulation at the Pb site, improving the overlap and hybridization of bonding orbitals. A familiar phenomenon occurred with Pb p orbitals in Pb SACs interacting with C p orbitals in ^*^CO [128]. With S dopants, the structure was binding strongly to ^*^CO, which resulted in poisoning of the catalyst. Coincidentally, the introduction of vacancy defects near the Pb sites can push away the hybridized orbitals from the Fermi level, resisting the overtight binding of ^*^CO and thus effectively allowing the CO_2_RR to proceed. The result underscores the synergistic modulation achieved by S doping and vacancy defect introduction, leading to moderate intermediate binding on Pb sites. This blueprint could be broadly applicable to other p‐block SAC systems, emphasizing the growing importance of defect and dopant engineering in advancing p‐block metal catalysis.

An expanding body of research supports the promise of *p*‐block metal single‐atom catalysts (SACs), demonstrating the importance of adjusting electronic structures and local coordination to maximize catalytic performance. Promising strategies, such as dual‐atom/triple‐atom catalysts (DAC/TAC), vacancy engineering, and targeted doping, are paving the way for future advancements in this area. Particularly noteworthy is the rising interest in p‐block MN_x_ moieties, which possess a unique ability to break traditional scaling relationships, offering new pathways for catalytic design. However, to transition SACs from academic interest to viable technologies, the field must progress beyond theoretical models and short‐term performance metrics. Greater emphasis is needed on operando characterization and long‐term stability benchmarks.

In our view, the most significant opportunity lies in exploiting the flexibility of p‐orbitals, the synergistic effects of defects, and non‐traditional back‐donation mechanisms to develop SACs that transcend traditional transition metal analogues, rather than merely replicating them. That said, SACs still face several fundamental challenges, including limited active site density, poor long‐term stability, and difficulties in scalable synthesis for industrial use. These challenges are especially common in harsher reactions like ORR or H_2_O_2_ production. Often these burdens would lighten with better atomistic and mechanistic insights. The molecular‐level insight into the dynamic evolution of active sites to deactivation is not yet well understood [129]. The limitation originates from the complex three‐phase interface, which DFT struggles to model. Despite these challenges, other avenues of electrocatalyst design have accepted the challenges. To overcome these limitations, researchers often introduce complementary or supporting atoms through doping strategies to enhance material performance and durability.

## 4 Doped Systems

One of the most versatile strategies for adjusting catalytic properties is doping, which allows for precise modulation of electronic structure, oxidation states, and hybridization. A recurring theme across high‐performing electrocatalysts is that their success stems less from the intrinsic properties of the host and more from the way dopants reshape surface reactivity. In this context, *p*‐block metal dopants are especially intriguing because they introduce new electronic degrees of freedom, beyond what d‐block doping typically achieves, while also offering low cost and abundance.

**FIGURE 13:**
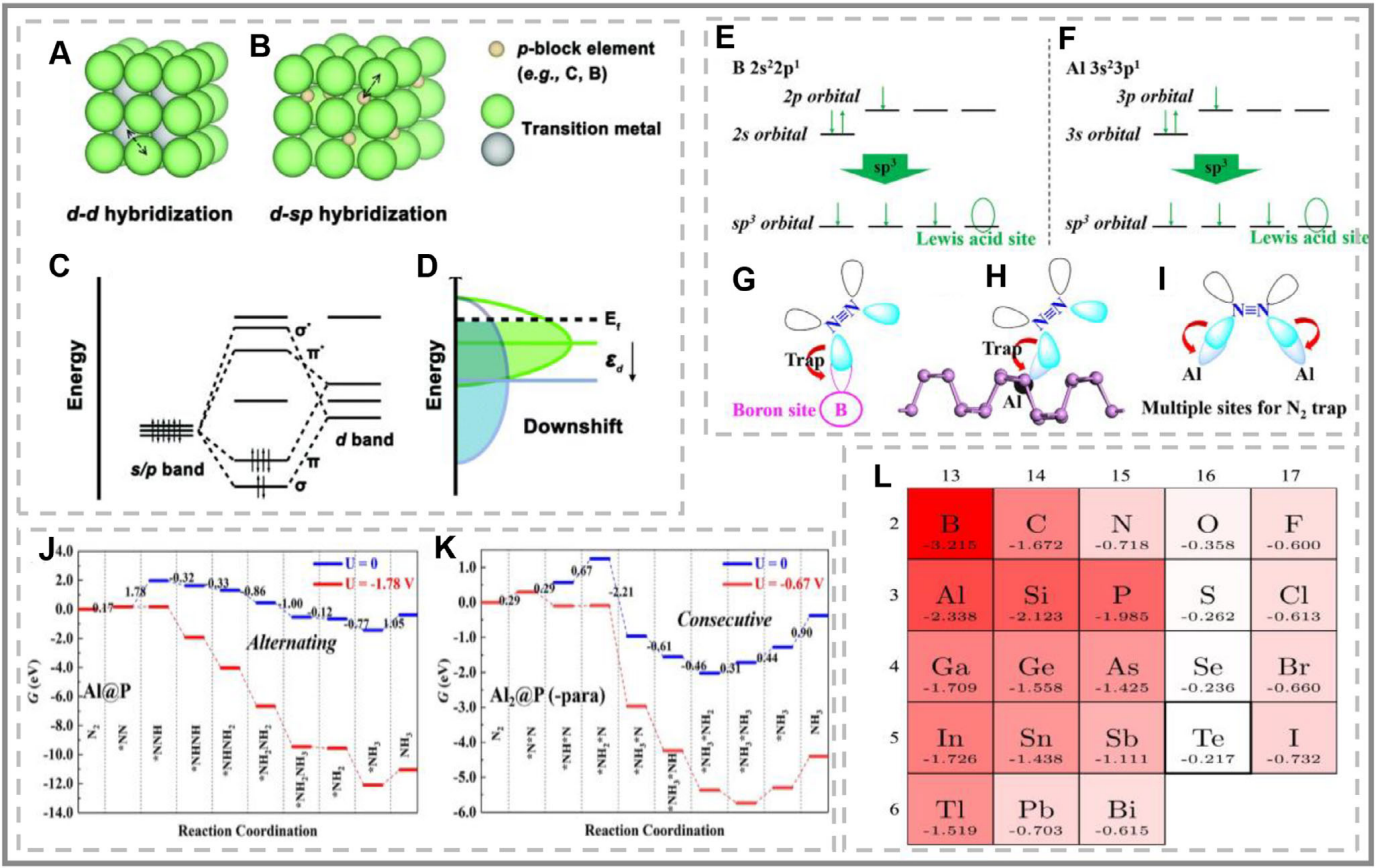
A simplified model of an alloy containing d‐d (A) and d‐sp (B) orbital hybridization. Schematic illustration of d‐sp orbital hybridization (C) and the induced effects on the d‐band of the transition metal (D). (A–D) Reproduced with permission [18]. Copyright 2022, The Royal Society of Chemistry. The similar valance electronics between B (E) and Al (F). (G–I) the N_2_ adsorption on single B/Al site with varying Al sites. NRR free energy on (J) AL@P and (K) AL_2_@P along the enzymatic pathway. (E–K) Reproduced with permission [134]. Copyright 2021, Elsevier B.V. (L) Calculated adsorption parameters for horizontal position of O_2_@X‐MoTe_2_: adsorption energy *E_ad_* given in eV. Reproduced with permission [135]. Copyright 2024, Elsevier Ltd.

Recent works have also focused on the synergistic effects of *p‐d* orbital hybridization for catalytic performance (see Figure 13A,B). The p‐d orbital hybridization is a well‐understood approach to improving a catalyst's performance. As the name implies, by bonding a p and d block metal together, the electronic properties of catalysts can be manipulated (see Figure 13C,D). This improves performance in three ways: the lowering of the d‐band so it is more inline with the reactants' p‐orbitals, an increase in electron density near the Fermi level, allowing for faster electron transfer [126], and the tuning of the molecular orbitals to improve stability of intermediate states [130]. Therefore, when available, p‐d hybridization is sought after. For example, hybridization COMPET with *p*‐orbitals can suppress Pt dissolution in ORR, while simultaneously tuning adsorption energies of intermediates [131, 132]. Recent *p–d* orbital hybridization strategies have primarily targeted noble metal catalysts, with more exotic materials remaining relatively underexplored [26, 133]. Here, we will specifically examine *p*‐block metal dopants and their influence on catalytic performance.

### 4.1 Low Dimensional Materials

Al is a common dopant as it is an effective active site on 2D materials. Z et al. (2022) discussed how Al‐doped phosphorene is an outstanding NRR catalyst [134]. Typically, 2D phosphorus (P) surfaces are poor NRR catalysts due to a weak activation of N_2_ and an inability to release NH_3_ [136]. Therefore, Al was introduced as an active site and sp^3^‐hybridised with neighboring P structures after the success of a B‐based catalyst (see Figure 13E–I). The resulting change in electronic properties allows Al to act as a Lewis acid, which accepts N_2_’s one‐pair electron and promotes binding. However, multiple Al dopants can improve performance further (similar to DAC and TAC). Specifically, dual atom doping is highly beneficial for weakening the N─N bond and activation [134]. Both single and dual Al atom dopants improve the selectivity and thermal stability of the 2D material, exceeding the anticipated N_2_RR performance (see Figure 13J,K). The overpotential of the leading dual Al atom catalyst was 0.15 V, and NRR occurred under ambient conditions. Therefore, p‐block dopants are not only corrective modifiers but can function as true active sites. The implication is that the careful positioning of multiple dopants might be just as significant as the metal selection, highlighting the promise of dual‐ and triple‐atom catalysts.

Doping studies on 2D molybdenum ditelluride (MoTe_2_) further reinforce the diversity of the p‐block effect [135]. Systematic screening of 22 p‐block dopants revealed clear periodic trends: smaller covalent radii favored in‐plane sp^2^ hybridization, while larger dopants favored sp^3^ bonding, with strong impacts on O_2_ adsorption (see Figure 13L) [137]. A general exception to this radius trend was Bi and Pd [135]. Both elements were shown to exhibit high charge transfer [36]. Pb‐ and Bi‐doped sheets, specifically O_2_@Pb‐MoTe_2_, exhibited strong adsorption and significant charge transfer between the O_2_ molecule and the dopant. A deeper orbital effect, not addressed in this study, was observed, highlighting that radius was not the only contributing factor. Further investigation of these findings may uncover additional outliers that could facilitate unconventional catalytic pathways.

Another novel material identified in recent years was the porous PtTeRh nanorods, which are designed from 1D building blocks [138]. The unique material has high ORR kinetics and longevity performing in a membrane electrode assembly at 1023.8 W g^−1^ over 240 h with little voltage loss. It far outperformed the commercial Pt/C catalyst under extreme cell operating conditions. Even after 30 000 cycles, the material only lost 7.5% in mass activity. Experimental and computational evidence suggested that the uneven surface of intersecting 1D planes provided a rich interfacial region with abundant active sites for oxygen reduction. Furthermore, the Te‐5*p* orbitals facilitated stable electrocatalytic activity over extended ORR cycles by shielding the weakly bound Pt and Rh d orbitals.

### 4.2 Oxides & Enhance Durability

Metal oxide phases are highly stable and cheap, which is desirable for a range of chemical reactions. However, their activity and performance can be lacking. Here, Bi can be used to dope the surface and enhance their performance. As mentioned before, SnO_2_ is an effective p‐block metal‐based electrocatalyst for CO_2_RR when its Sn species is oxidized and does not revert to its metallic state [78, 139, 140]. When compared to pure SnO_2_ and Pt‐doped SnO_2_, Bi‐SnO_2_ exhibited higher stability and activity, ensuring a high Faradaic efficiency of 70% (for formate) until the current density reached 300 mA cm^−2^ [141]. Bi substitution induces similar adsorption effects to Cu dopant, but is still inferior to Cu, as Cu has a larger current density ranging in 100–500 mA cm^−2^. Another example involves using a Bi‐doped Co_3_O_4_ nanoflake to oxidize glucose [142]. Utilizing the p‐d orbital and Bi to interact with the 2p orbitals of O, N, and C, the material exhibited efficient glucose oxidation (of 1.25 V for a current density of 10 mA cm^−2^). The material required 1.39 V vs. RHE to achieve a current density of 50 mA cm^−2^, outperforming commercial alternatives [142]. The Bi‐Co_3_O_4_ material also exhibited fast charge transfer, changing the double‐layer capacitance to increase the active surface area of the catalyst. The electron cloud around the Co_3_O_4_ material was reduced and relocated to the Bi^3+^ species present on the surface. Bi^3+^ and its other states were present in various works. Specifically, for Bi^3+^, the lone pair of electrons activates catalytic sites through stereochemical effects [143]. In oxygen reduction, the 6s orbital near the Fermi level interacts with the 2p orbital, producing strong charge transfer. The Bi^3^^+^ state in BiMn_2_O_5_ distorts the Mo‐O distance, resulting in improved adsorption of intermediate species.

Al is resilient to high temperatures and pressures while also being utilized in a broad range of reactions. Lv X‐W (2020) et al. discuss a 3D Ni foam‐supported urchin‐like Al‐doped Co_3_O_4_ nanospheres rich in surface oxygen vacancies (Al‐Co_3_O_4_/NF) [144]. The incorporation of Al atoms ultimately adjusted the electronic and structural features of the catalyst, while simultaneously increasing surface oxygen vacancies. These vacancies facilitated the activation of N_2_ molecules, enhancing their reduction to NH_3_. The synergy between the Ni foam and Al dopant demonstrated thermal stability by safeguarding the Co_3_O_4_ nanostructures against dissolution [145]. The Al^3+^ forms an oxide shell that prevents breaking down the charge carrying foam and stabilizes the Co nanoparticles [146]. This synergy between the Al dopant and Ni foam substrate underscores the potential of Al‐modified materials in advancing robust and high‐performance catalytic systems for ammonia synthesis and related applications.

### 4.3 Doping for CO_2_RR and ORR Selectivity and Electron Transfer

**FIGURE 14:**
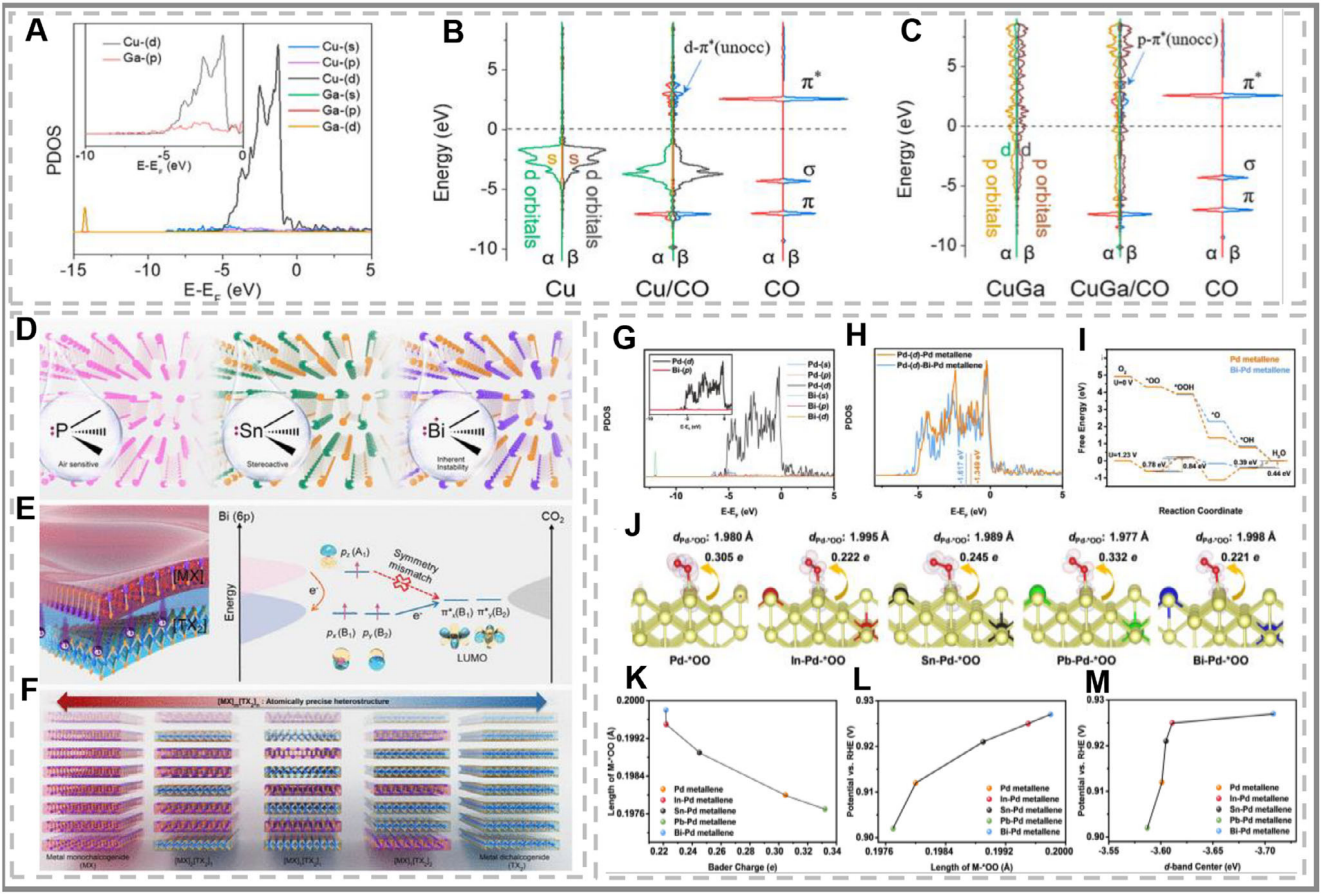
Electronic and structural effects of p‐block metal doping on electrocatalysis. (A–C) Projected density of states (PDOS) analyses show how Ga doping in Cu induces p–d orbital hybridization with CO intermediates, enhancing ^*^CO adsorption and promoting C_2_^+^ product formation in CO_2_RR. (A–C) Reproduced with permission [147]. Copyright 2023, American Chemical Society. (D–F) Schematic illustrations of structural challenges and opportunities in p‐block monochalcogenides: instability of BP‐like structures, interlayer charge transfer in misfit superlattices, and tunability via sublayer ratios to optimize orbital alignment for CO_2_ activation. (D–F) Reproduced with permission [10]. Copyright 2025, American Chemical Society. (G–M) Electronic structure and reaction pathway analysis of Bi‐doped Pd metallenes: PDOS shows Bi p‐orbitals interacting with Pd d‐orbitals, shifting the d‐band center, and modifying charge transfer. Free energy diagrams, charge density differences, and Bader charge analyses demonstrate that p‐block doping modulates oxygen binding, breaking scaling relations and enhancing ORR activity. (G–M) Reproduced with permission [148]. Copyright 2024, Wiley‐VCH.

To truly understand the synergistic effects of p‐block metals, a holistic approach is necessary to connect electronic redistribution with catalytic performance. The study by L. et al. (2023) focused on how Ga, Al, and Ge dopants affected Cu in advancing CO_2_RR [147]. Ga‐doped Cu surfaces were highlighted for their ability to stabilize Cu^+^ species, thereby enabling C_2_^+^ product formation. Despite this advantage, the pathway remained highly challenging owing to complicated electron transfer and limited ^*^CO adsorption, which favored HER activity. The p‐d hybridization between Ga and Cu led to a redistribution of electrons (see Figure 14A–C). The energy levels of Cu 3d/Ga 4p orbitals and CO π^*^ orbitals are strongly overlapped, leading to the formation of d‐π^*^ and p−π^*^ bonds. A broad dispersity was observed for the p−π^*^ orbitals above the Fermi level, suggesting the enhancement of interactions between the active site and the CO molecule after doping of Ga (see Figure 14C). The result was additionally validated when the π orbital of CO in the gas phase moved below the Fermi level after binding to the CuGa surface. The outcome of the redistribution resulted in a Faradaic efficiency of 81.5% at a current density of 0.9 A/cm^2^ and a potential of ‐1.07 V vs. RHE. Importantly, CuAl and CuGe showed similar p–d interactions, outperforming pure Cu but not reaching Ga's selectivity. This suggests that Ga's electronic configuration offers a uniquely favorable balance between CO stabilization and suppression of HER, a design principle that should guide future dopant selection rather than relying on empirical screening.

Doping engineering extends well beyond Cu. L1_0_‐PtM (M = Fe, Co, Ni, and so on) intermetallic nanocrystals are chemically unstable but highly active catalysts [149]. Their synthesis requires high‐temperature annealing, which leads to poor particle generation at the gram‐scale. However, by introducing a Sn, Ga or In dopant, the surface energy is weakened to promote the ordering process of the PtM alloy catalyst. These dopants can lower the annealing temperatures (200°C reduction), thus enabling the preparation of Pt‐rich intermetal nanocrystals at large scales. The developed L10‐Ga‐PtNi/C catalysts display outstanding performance in H_2_–air fuel cells under both light‐ and heavy‐duty vehicle conditions. Under heavy‐duty vehicle conditions, the 40% L10‐Pt_50_Ni_35_Ga_15_/C catalyst reached a current density of 1.67 A cm^−2^ at 0.7 V and sustained 80% of its activity after 90 000 cycles. These results exceeded the standards defined by the United States Department of Energy and demonstrated its standing as one of the best cathodic electrocatalysts for proton‐exchange membrane fuel cells. However, Pt remains expensive, so other earth‐abundant compounds have been investigated.

Manganese oxides are attractive alkaline ORR catalysts due to their low cost, abundance, and structural versatility, yet they suffer from poor conductivity and weak oxygen binding [150, 151]. Qin et al. (2023) addressed this by incorporating Ga, Al, and In ions through a one‐step hydrothermal synthesis [152]. Here, p–d hybridization within X–O–Mn (X = Ga, Al, In) enriched reaction sites and enhanced intermediate adsorption. Ga in particular modulated the Mn d‐band center, lowering the barrier for O–O bond cleavage. The resulting Ga–MnO_2_ exhibited a maximum power density of 86 mW cm^−2^ with a Tafel slope of 81.2 mV dec^−^^1^, rivalling Pt/C in activity while matching its durability [152].

The common thread across these studies is that *p*‐block dopants act not merely as passive modifiers but as active participants in orbital engineering. By reshaping electronic distributions and tuning adsorption energetics, they can break conventional scaling relationships and introduce new catalytic pathways. The field should move beyond opportunistic doping toward a predictive framework that links dopant electronic structure, orbital overlap, and host lattice flexibility. In this respect, *p*‐block monochalcogenides (MX) represent an up‐and‐coming platform, as their intrinsic orbital softness and layered structures are ideally suited for synergistic p–d interactions.

A MX sublayer consists of *p*‐block (e.g., Bi, Sn, and Pb) or rare earth metals with chalcogen elements (see Figure 14D–F). These structures exhibit high active surfaces for CO_2_RR [10, 14, 153]. Simultaneously, the TX_2_ metallic sublayer acted as a current collector, promoting efficient electrocatalysis at high current densities [154, 155]. The resulting interlayer electronic coupling within the misfit layers induces non‐invasive doping and local charge redistribution. The electron shift modulates the orbital occupation of *p*‐block metal active sites, reducing localized electron density and enhancing orbital overlap with adsorbates. Therefore, the ability to fabricate naturally occurring hierarchical vdW heterostructures provides a versatile platform for investigating the impact of interlayer charge transfer on stability and catalytic activity by tailoring their sublayer composition. According to the Bader charge analysis, the dominant contribution to charge donation came from the Bi atoms in BiS [10]. In contrast, Sn‐ or Pb‐based metal monosulfides exhibit lower charge transfer from the MX sublayer to the TX_2_ sublayer than Bi, highlighting the strong electronic interaction between BiS and TaS_2_. The consequence of this transfer is the stabilization of higher Bi valence states (Bi^3+^). Partial density of states analysis shows that the Bi Pz orbital decreases significantly near the Fermi energy after forming the superlattice, leading to a more delocalized electron density and a broader PDOS distribution near the Fermi energy. Additionally, electron transfer raises the energy of the Bi Pz orbital and redistributes electrons from the Pz orbital to the Px and Py orbitals, significantly minimizing internal lattice distortions in BiS as well as impacting the CO_2_RR performance. The material demonstrated high Faradaic efficiency, exceeding 90% across a –0.6 to –1.1 V vs. RHE range, while retaining a current density of about 60 mA cm^−2^.

Besides the beneficial catalytic performance, MX synthesis and stability are the main limitations for practical applications. Strong interactions between these monolayers and water molecules led to fast degradation [145]. While some mitigation approaches had been reported, further efforts were needed to enhance their longevity [156]. Scalability is a primary cause of concern. Current synthesis routes, such as laser etching and exfoliation, are labor‐intensive and are unsuitable for large scale productions. Reproducibility remained an issue for these materials because subtle changes in precursor conditions significantly affected their properties [153]. However, other 2D materials also hold potential.

In addition to the doping strategies discussed above, incorporating isolated single atoms into a host metal to construct single‐atom alloys (SAAs) represents another promising engineering approach for tuning electrocatalytic performance. As the concentration of p‐block dopants in the host metal decreases to the atomic limit, single‐atom alloys can be formed. This strategy has emerged as an effective means of tailoring the localized microenvironment surrounding metal active sites, thereby modulating their electronic structures and catalytic properties [157]. In 2024, Xie et al. introduced doping single p‐block metal atoms (In, Sn, Pb, and Bi) onto ultrathin Pd metallenes for catalyzing the ORR [148]. Through precisely controlling the Bader charge transfer between the Pd surface and oxygenated intermediates (^*^OO) via p–d orbital interactions, the authors achieved a downshift in the Pd d‑band center (see Figure 14G,H). The effect weakened the adsorption strength of O intermediates, a fundamental kinetic hurdle for the ORR (see Figure 14I) [148, 158]. Among the dopants, BiPd metallene delivered standout mass activities of 11.34 A mg^−^^1^ at 0.90 V vs. RHE and 0.86 A mg^−^^1^ at 0.95 V vs. RHE under alkaline conditions. This result outperforms any previously reported Pd based ORR catalyst [148]. In practical zinc–air battery testing, the Bi doped catalyst exhibited a high open‐circuit voltage of 1.55 V and impressive durability, sustaining 760 h at 10 mA cm^−2^. Theoretical modelling suggests that Bi's inclusion optimally reduces Bader charge transfer and elongates the Pd–^*^OO bond, fine‐tuning adsorption energetics to promote faster ORR kinetics (see Figure 14J–M) [159]. Overall, this work showcases how atomic‐level modulation of electronic structure through p‐block doping can dramatically elevate ORR activity in Pd metallenes, highlighting a promising path for cost‐effective, high‐performance electrocatalysts.

In summary, the use of p‐block metal dopants has demonstrated a diverse and adaptive capacity to modulate the catalytic activity of their host material. The mechanism is rooted in the p‐d orbital hybridization and geometric tuning. The p‐block metal dopants alter the local electronic and physical properties of the active site while maintaining low costs. Importantly, these effects were highly context‐dependent, indicating that a targeted strategy was necessary when optimizing performance for a specific catalytic process. However, the main bottleneck is not activity but synthetic reproducibility and site identification. This is one of the main reasons why dopants remain underexplored in p‐block catalysis.

Most studies stop at computational screening or isolated case studies, with little experimental validation or scalable synthesis. Stability is another blind spot. Dopant migration and clustering under operating conditions may erase the very advantages that make doped materials attractive. In our view, the next frontier lies in combining doping with defect engineering, vacancy modulation, and even dynamic re‐doping strategies to stabilize the active sites. Furthermore, heavier p‐block elements (Sb, Bi, Pb) remain almost untouched as dopants, despite their demonstrated ability to alter charge transfer in unexpected ways.

Taken together, p‐block doping strategies are not just incremental tweaks to host systems but a conceptual opportunity to redefine how electronic structure is controlled in electrocatalysis. By moving beyond noble‐metal hosts and embracing defect‐rich 2D materials, multi‐dopant configurations, and heavier p‐block elements, the field can unlock a genuinely distinct pathway for catalyst discovery.

## 5 Anisotropic‐Structured Materials

Alloying is a natural extension of dopant‐based strategies. Homogenous materials were commonly produced using main group metals. Within these alloy frameworks, the metals interacted cooperatively, which intensified the p‐d and d‐d orbital interactions between the two elements involved [160, 161]. Additionally, alloying introduces lattice strain, morphological changes, and ensemble effects, all of which influence surface adsorption and reactivity [162]. One challenge of doped materials is precisely controlling the local structure and generating stable active sites under electrolysis conditions [147]. Here, alloys occupy a broader design space for tuning reaction pathways, stabilizing active phases, designing multifunctional sites, and modifying material morphology [31, 163]. As such, p‐block alloys have emerged as a versatile heterogeneous catalyst for various reactions.

### 5.1 Pt Group & p‐Block Alloys

**FIGURE 15:**
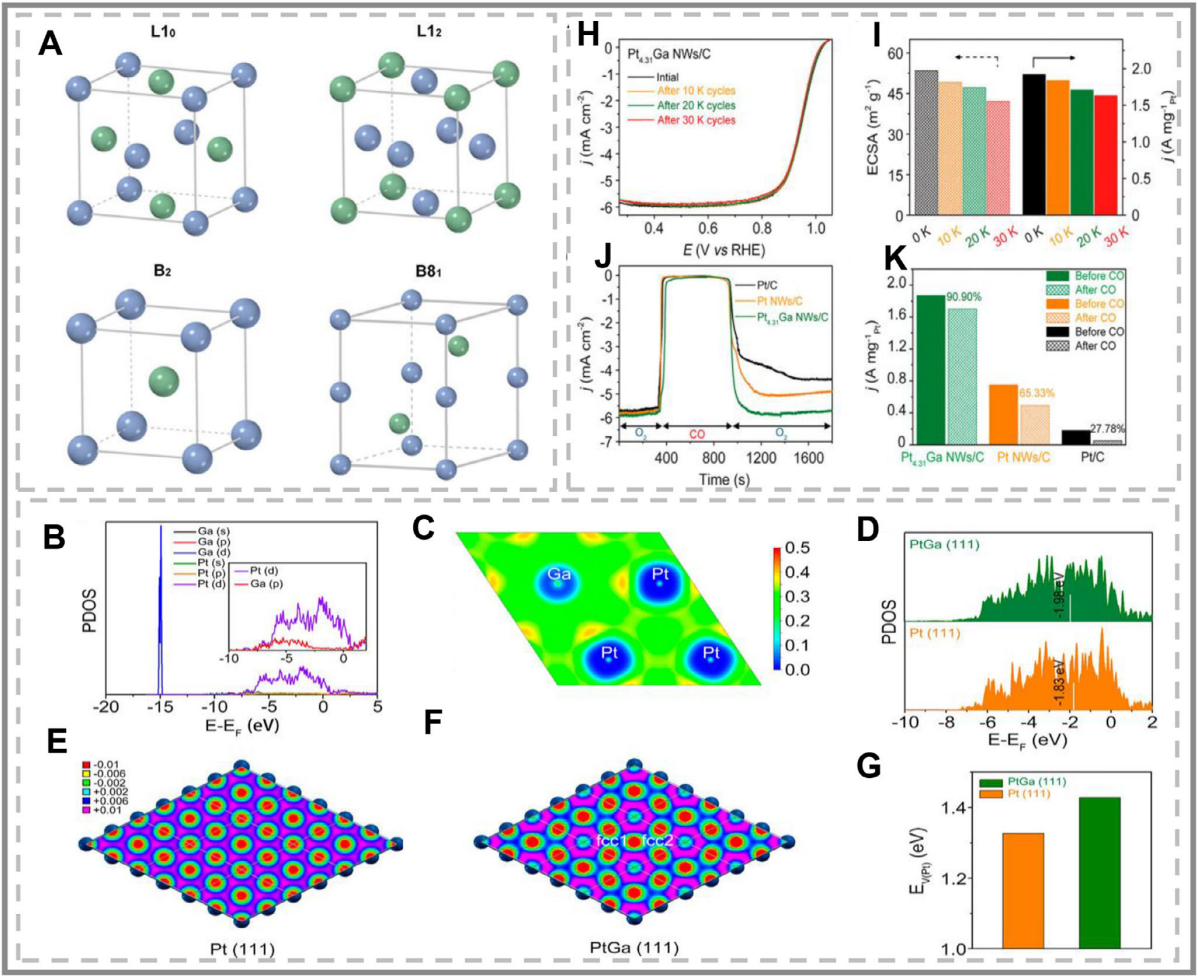
(A) Illustration of common intermetallic alloy structures. (B) The PDOS of PtGa alloy slab showing the Pt d‐orbital and Ga p‐orbital. (C) The electron localization function analysis was mapped for the second atomic layer in the PtGa alloy slab. (D) The plots of projected d‐density of states (PDOS) of surface Pt atoms for Pt and PtGa alloy slabs. The white lines show the corresponding d‐band centers. (E,F) The differential charge distributions for Pt (E) and PtGa alloy slabs (F). (H) Pt vacancy formation energies (EV(Pt)) for Pt and PtGa alloy slabs. (A) Reproduced with permission [21]. Copyright 2024, Wiley‐VCH. (H) ORR polarization curves of Pt_4.31_Ga NWs/C catalyst before and after different potential cycles. (J) The changes in ECSA and mass activity of Pt_4.31_Ga NWs/C catalyst before and after different potential cycles. (l) *i–T* curves recorded under the alternatively bubbling of O_2_ or CO for the catalysts at the potential of 0.1 vs. RHE. (K) The comparisons in mass activity at 0.9 vs. RHE before and after CO‐tolerance measurements. (B–K) Reproduced with permission [26]. Copyright 2019, American Chemical Society.

Alloys combining p‐block metals with PGMs have demonstrated synergistic effects for catalysis. Generally, for platinum group and p‐block metal alloys, due to the higher electronegativity of platinum group metals [164], electron transfer occurs through the p‐block metals into the PGM. This results in a significant shift of the d‐band center, consequently modifying the adsorption energy of reaction intermediates and enhancing the catalytic activity. Additionally, the large difference in atomic radius between the two types of atoms allows for tuning of the structural properties (see Figure 15A). The resulting amorphous structure is difficult to maintain using only d‐block metals. Therefore, the structural diversity of the d/p block alloys can allow for unique catalytic morphologies.

The use of p‐block metals also offered the benefit of low melting points, which made intermetallic synthesis more manageable. Incorporating low‐melting metals, especially Sn, lowered both the activation energy and the metallic bond strength in PtM (M = Fe, Co, Ni, Cu, Zn) systems, leading to improved ordering during alloy formation [149]. The resulting process causes fast formation/diffusion of low‐coordinate surface free atoms, which are ideal for high catalytic activity and stability. The most effective catalyst for ORR was the Ga‐doped PtNi/C composition, which exhibited remarkable performance under the demanding conditions of heavy‐duty vehicles. It delivered 1.67 A cm^−2^ at 0.7 V and retained 80% of the current density for over 90 000 cycles.

A notable example was demonstrated by Lei et al. (2019), who constructed a high‐performance PtGa alloy for the ORR that featured an unconventional p‐d hybridization interaction [26]. Interestingly, the Pt‐Ga interactions enacted through their p‐d bonds weakened the materials' binding strength for oxygenated species (see Figure 15B–F). This weakening generated a more favorable energy profile than pure Pt and enhanced the overall ORR performance [31]. Durability and resistance to oxidation are common issues with non‐transition metal catalysts [165]. The PGMs’ resilience to oxidation and degradation is a strong reason for the dominance of these catalysts. However, the PtGa alloy was shown to improve oxidation resistance and structural stability. Surface vacancies, which are ideal for active sites but less stable, were more common on the PtGa surface than on pure Pt (see Figure 15G) [26, 166]. The increased vacancy sites suggest that an increase in the vacancy formation energy, which is due to the elevated reduction potential of the surface Pt. Electron accumulation near the Pt atoms gave rise to the change, driven by a resulting p–d hybridization with Ga.

Additionally, the leaching of Ga is suppressed through the same strong p‐d hybridization interaction, which causes the diffusion of Ga atoms to the exterior. Based on these results, the resulting PtGa alloy was around 12.1 times more active than commercial Pt/C catalysts [26]. Furthermore, the catalyst was over five times more durable than Pt/C catalysts (see Figure 15H–K) [167, 168]. A similar study also found an increased performance with PtGa and PtIn alloys, but focused on saltwater electrolysis [167]. Presumably, the electronic structure of the group 13 p‐block metals is ideal for metal alloys because the d‐band center is shifted toward a favorable position to enhance catalytic performance. Their electronic structure shifts the d‐band center to a more optimal position, thereby improving performance.

Despite these successes, Pt‐based alloys remain constrained by cost and stability concerns. A persistent limitation is the leaching of the non‐noble alloying element, which undermines long‐term durability [169, 170, 171]. For instance, Pt–Ni alloys have been widely studied as potential replacements for Pt/C, often surpassing it in activity but falling short in operational lifetime [172]. X. et al. (2019) showed that doping Pt‐Ni with indium increases the lattice atom diffusion barrier and decreases the surface energy [173]. This material decelerates the leaking of internal Ni atoms and promotes the stability of the active sites. However, the same modification also shifted the band structure away from the optimal, slightly weakening intrinsic activity. Even so, the ternary Pt–In–Ni system achieved a mass activity of 0.76 A mgPt^−^^1^, four times that of conventional Pt/C (0.20 A mgPt^−^^1^). These findings highlight a central dilemma: alloying can dramatically enhance activity and sometimes durability, but it often introduces trade‐offs in band alignment and selectivity.

Taken as a whole, these studies underscore both the potential and the limitations of Pt‐based p‐block alloys. While Ga and In doping clearly enhance stability and tune intermediate binding, they do not resolve the cost barrier of PGMs. Therefore, the driving value of Pt‐based p‐block alloys was rooted in their ability to engineer p–d hybridization, thereby enhancing durability and breaking traditional scaling relations. The next step is to translate these insights into PGM‐free systems where cost and sustainability align with catalytic performance.

### 5.2 Non‐Transition Metal Alloys

**FIGURE 16:**
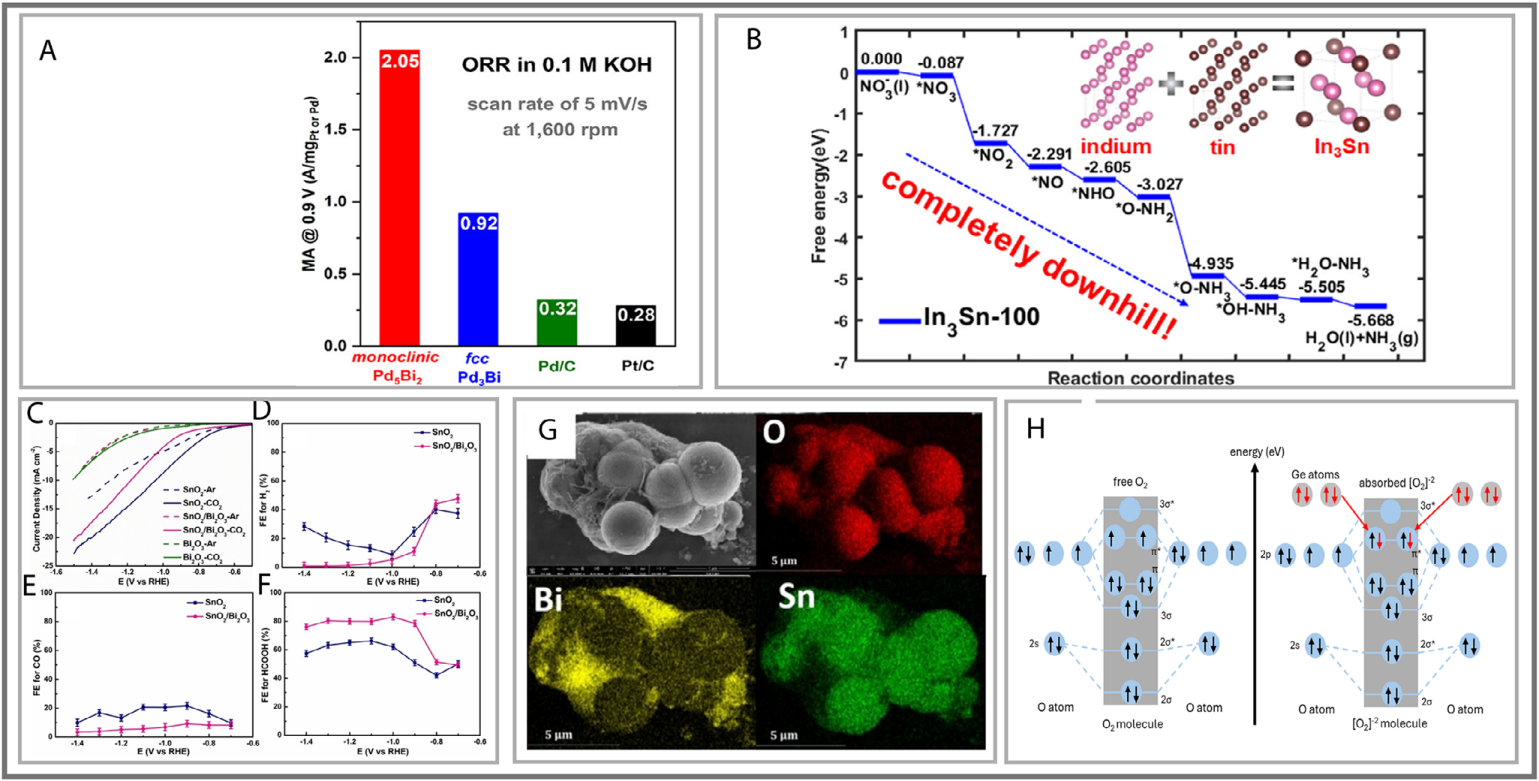
(A) Structure of monoclinic and fcc Pd‐Bi alloy with performance metrics compared to Pt/C and Pd/C. Reproduced with permission [168]. Copyright 2021, American Chemical Society. (B) Free energy diagram of NRR using In_3_Sn catalysts. Reproduced with permission [174]. Copyright 2023, American Chemical Society. (C) CO_2_RR activities of the prepared catalyst in Ar‐ (dash line) and CO_2_‐saturated (solid line) 0.1 m KHCO_3_ electrolyte. Faradaic efficiencies (FE) of H_2_ (D), CO (E), and HCOOH (F). (G) SEM image and the corresponding EDS elemental mappings of SnO_2_/Bi_2_O_3_. (C–G) Reproduced with permission [175]. Copyright 2021, Wiley‐VCH. (H) The Kohn–Sham orbital of the isolated and activated O_2_ molecule. The model of activated O_2_ was adopted from the freestanding germanene monolayer. Reproduced with permission [176]. Copyright 2024, AIP Publishing.

Identifying potential alternatives to PGMs is essential for advancing several electrochemical reactions. Redirecting from PGMs is important to improve the affordability of energy technologies. Therefore, several works have focused on p‐block alloys. The work by Zhou et al. (2021) focused on alkaline ORR and utilized the unique morphology of p‐block alloys to enhance catalytic ability [168]. Their work aimed at PdBi catalysts and discovered a stable metal configuration that enhanced catalytic performance. Through altering the types of Pd precursors, the monoclinic structured Pd_5_Bi_2_ and the conventional face‐centered cubic (fcc) counterpart can be precisely synthesized with comparable sizes and morphologies [168]. The monoclinic Pd_5_Bi_2_ nanocrystal greatly enhanced electronic interaction, which resulted in stronger antioxidant capabilities than the fcc Pd_3_Bi structure. The monoclinic catalyst's unique structural configuration enabled a distinct ratio of Bi^0^ and Bi^3^^+^ species, which resulted in greater chemical stability than that of its fcc counterpart (See Figure 16A). When comparing their catalytic performance, both the fcc and monoclinic PdBi catalysts outperformed commercial Pt/C [167, 168]. Both PdBi catalysts preferred the 4e‐ ORR pathway, producing current densities of 5.8 mA cm^−2^ and a mass activity 7.3 times higher than Pt/C [168]. In general, the monolithic Pd_5_Bi_2_/C catalyst outperformed all materials cited and investigated in both catalytic performance and durability. The interesting structure and electronic distribution of these materials should stand as an excellent jumping‐off point for future research in this area.

X et al. (2020) demonstrated a universal and robust Bi_2_Te_3_ nanoplate electrocatalyst [177]. The material simultaneously reduced small molecules (like CO_2_, N_2_, and O_2_) with high selectivity and durability. For ORR, they achieved nearly complete selectivity for H_2_O_2_ between 0.2 and 0.6 V vs. RHE. During CO_2_RR in a NaHCO_3_ electrolyte, they delivered a partial current density of 24.2 mA cm^−2^ for HCOOH at –1.1 V vs. RHE with a Faradaic efficiency of 89.6%. For NRR, NH_3_ was produced with a Faradaic efficiency of 7.9% at –0.4 V vs. RHE in KOH. Despite the high performance in HCOOH and H_2_O_2_ generation, these represent the kinetically and thermodynamically easiest pathways in their respective reactions. However, they are still required, and a versatile catalyst is highly desirable. The origin of the high performance originates in the Te vacancies after electrochemical activation. The presence of Te vacancies significantly shifted the Bi‐6p bands closer to the Fermi level, thereby activating them. As a low‐cost and elegant approach, vacancy engineering held promise for improving both durability and catalytic performance and should be thoughtfully considered in the design of future p‐block metal electrocatalysts. However, the approach also carried the risk of degrading surface energy, which frequently leads to nanoparticle agglomeration and reduced dispersion.

Hanquing et al. focused on this problem and studied several alloys of In‐Sn, including In_3_Sn and InSn_3_ for NRR [174]. By alloying Sn with In, the selectivity of the ^*^NHO was higher than that of the monocrystalline In. For In_3_Sn(100) alloy, the free energy profile is all downhill, which is extremely rare in existing works (see Figure 16B). The InSn_3_(100) surface, in contrast, suffered from thermodynamically unfavorable conditions, showing positive adsorption energy for nitrate and a positive Gibbs free energy barrier for NO hydrogenation. Both alloys were still greater than either monocrystal. The alloys were also superior in suppressing HER than Pt‐based catalysts [167].

Another study conducted by Dr. Jianjian Tian and colleagues in 2021 introduced a heterostructure composed solely of p‐block elements, which demonstrated excellent catalytic performance in CO_2_ reduction (see Figure 16C–F) [175]. They synthesized a heterostructure with SnO_2_ clusters on a Bi_2_O_3_ matrix to support the maintenance of the Sn^4+^ species (see Figure 16G). A strong electric field was produced through the interaction between the interface of SnO_2_ and Bi_2_O_3_ as their Fermi levels were in contact. The electric field and electronic overlap indicated a fast transfer of electrons, enhancing CO_2_RR performance. Over the broad potential window of –1.0 to –1.4 V vs. RHE, the material sustained a field factor exceeding 76%, with a maximum of 90% observed at –1.0 V. The strong built‐in electric field at the heterointerface may be the most critical factor for the excellent HCOOH selectivity over SnO_2_/Bi_2_O_3_ composite. By constructing a specialized electron‐transfer interface, they effectively enhanced CO_2_ binding and activation and achieved improved stabilization of intermediates during CO_2_ electroreduction. According to these results, the formation of SnO_2_/Bi_2_O_3_ composite strongly enhances the catalytic activity of SnO_2_.

Germanene nanosheets are an attractive novel 2D material for catalytic reduction [176]. Germanene's unique honeycomb structure, mixed with sp^2^ and sp^3^ hybridization, possesses several distinctive catalytic properties [178, 179, 180]. However, their synthesis required a (111) metallic surface, which was often associated with the use of noble metals [181]. The substrate on which the 2D material is built stabilizes and regulates electronic and surficial activity via interlayer electron coupling [178, 182]. P et al. (2024) focus on germanene on Al(111) for ORR through a computational lens [176]. In comparison to other substrates, Al with s‐type orbitals exhibits hexagonal symmetry, which is very close to free‐standing germanene. Therefore, an Al substrate would enable simple large‐scale production at low cost. The study shows that the Al substrate provides the germanene a flexible backbone, which enables the 2D material to bend and deform, thus improving longevity. The surface stretching serves a catalytic function, as the O_2_ molecules adsorb on the surface, they are pulled apart following the 4e‐ ORR dissociation mechanism. A detailed investigation showed that the sp‐orbitals of the Al substrate hybridize with the sp‐orbitals of the surficial Ge atoms. This results in a flatter Ge p‐orbital, which improves the stability and interactions of O p‐orbitals bonded with Ge atoms (See Figure 16H). The change in orbital structure improves the transfer of electrons and the reaction rate. They achieved ORR performance that outperformed conventional Pt‐based catalysts in terms of rate‐determining activation energy and Gibbs free energy [183, 184].

Alloys and heterostructures composed of p‐block metals provided a versatile platform for tuning catalytic properties via structural and electronic engineering. Evidently, the synergistic effects between non‐transition metal p‐block metal alloys hold potential for advancing electrocatalysis. These systems unlock new pathways for optimizing activity, selectivity, and durability. However, more studies are required to study the diverse range of potential p‐block metal alloys.

## 6 Conclusion

### 6.1 Key Insights from P‐Block Catalysts

P‐block metal‐based electrocatalyst presents unique advantages compared to traditional platinum group metal systems. The inherent oxophilicity, weak hydrogen adsorption, and tunable valence states endow p‐block metal catalysts with exceptional selectivity in small‐molecule electroreduction reactions. Numerous morphologies and designs appeared across the literature, and their performance in CO_2_ and N_2_ reduction was compiled in Tables 4 and 5. However, the ORR field showed a significant lack of new research outputs despite some promising progress in selected publications (Table 6).

**TABLE 4:** The performance of recently reported p‐block metal‐based electrocatalysts for CO_2_RR.

Catalyst	Electrolyte	Product	Faradaic Efficiency (%)	Current Density (mA cm‐2)	Potential V vs. RHE	Longevity	Refs.
[BiS]_1_[TaS_2_]_1_	1 m KOH	Formate	90	110	−1.1	24 h	[10]
Activated Bi_2_Te_3_ NPs/C	0.5 m NaHCO_3_	Formate	89.6	31	−1.6 Ag/AgCl	10 h	[177]
Al–NC	0.1 m KHCO_3_	CO	98.76	309	−0.65	16 h	[114]
Bi nanoflakes	0.1 m KHCO_3_	Formate	100	4.8	−0.6	10 h	[20]
Bi nanostructure	0.5 m KHCO_3_	Formate	92	15	−0.9	30 h	[185]
Bi NPs/Bi_2_O_3_ NSs	0.5 m KHCO_3_	Formate	100	6.2	−0.86	24 h	[186]
Bi NWs @Cu foam	0.5 m NaHCO_3_	Formate	95	15	−0.69	12 h	[187]
Bi SAs/NC	0.1 m KHCO_3_	CO	97	5.1	−0.5	4 h	[188]
Bi/C NPs	1 m KOH	Formate	89.2	45	−3	90 min	[189]
Bi@NCFs	0.5 m KHCO_3_	Formate	95.7	11.4	−1	40 h	[190]
Bi_2_O_3_‐NGQDs	0.5 m KHCO_3_	Formate	97.4	18.2	−0.9	15 h	[191]
Bi_2_S_3_ nanoflower	0.5 m NaHCO_3_	Formate	90	4.8	−0.75	10 h	[91]
Bi‐CMEC	MeCN/[BMIM]BF_4_	Formate	95	95	−1.95 V vs. SCE	4 h	[192]
Bi‐PMo NSs	0.5 m NaHCO_3_	Formate	93	30	−0.86	10 h	[193]
CADF indium	0.5 m KHCO_3_	Formate	86	5.8	−0.86	3 h	[64]
Ce‐doped Bi(0) nanoparticles	0.1 m KHCO_3_	Formate	97.2	13.7	−1.1	48 h	[50]
Commercial Sn	1 m KHCO_3_	Formate	93.3	51.7	−2.2	50 h	[194]
Cu/Bi_2_S_3_	0.5 m KHCO_3_	Formate	94	∼6	−0.8	100 h	[195]
Cu–In alloy	0.1 mm KHCO_3_	Formate	75	0.3	−0.6	/	[196]
Cu‐incorporated SnO_2_	0.1 m KHCO_3_	Formate	80	500	−1	5 h	[141]
Etched Bi_2_O_3_ nanorods	0.5 m KHCO_3_	Formate	99.2	7.5	−1.5 V vs. SCE	20 h	[93]
Fluorine‐doped tin oxide	1 m KHCO_3_	Formate	95	100	−1	7 days	[140]
Ga‐doped Cu	1 m KOH	C2+ products	81.5	0.9	−1.07	12 h	[147]
In	0.1 m KHCO_3_	Formate	88.4	5	−1.48 vs. NHE	na	[69]
In_2_O_3_–rGO	0.1 m KHCO_3_	Formate	84.6	22.2	−1.2	10 h	[197]
InA/NC	0.5 m [Bmim]PF6	CO	97.2	39.4	−2.1 V vs. Ag/Ag+,	24 h	[119]
In–N_3_–V	0.5 m KHCO_3_	CO	95	2.3	−0.57	14 h	[116]
In–N_4_	0.5 m KHCO_3_	CO	80	20	−0.47	14 h	[116]
In–N–C	0.5 m KHCO_3_	Formate	80	6.8	−0.79	20 h	[198]
InO_x_ nanoribbons	0.5 m NaHCO_3_	CO	91.7	15.2	−1.05	20 h	[124]
In–Sn NPs	0.1 m KHCO_3_	Formate	78.6	9.6	−1.2	12 h	[199]
Mn–In_2_S_3_	0.1 m KHCO_3_	Formate	86	20.1	−0.9	8 h	[12]
mp Bi nanosheets	0.5 m NaHCO_3_	Formate	100	18	−1.1	12 h	[49]
mp‐Bi	0.5 m NaHCO_3_	Formate	100	15	−0.9	12 h	[66]
mp‐SnO_2_	0.5 m NaHCO_3_	Formate	83	14	−0.9	20 h	[200]
m‐SnO_2_	0.1 m KHCO_3_	Formate	75	10.8	−1.15	16 h	[201]
NTD‐Bi	0.5 m KHCO_3_	Formate	100	42	−0.85	48 h	[202]
Pb	0.1 m KHCO_3_	Formate	97.4	5	−1.63 vs. NHE	/	[69]
Pb dendrite	1 m KHCO_3_	Formate	97	7.5	−0.99	100 min	[203]
Pb‐MOF	0.1 m KHCO_3_	Formate	96.8	1.9	−0.88	5 cycles	[204]
Pb‐N_2_SV	0.5 m KHCO_3_	CO	97.3	11.74	−0.47	33 h	[126]
POD‐Bi	0.5 m KHCO_3_	HCOOH	95	55.6	−1.16	1600 s	[13]
R‐In_2_O_3_	0.1 m KHCO_3_	Formate	91.2	12.67	−1.27	80 h	[202]
Sb_0.1_Sn_0.9_O_2_	0.5 m KHCO_3_	Formate	92	1000	−0.9	200 h	[85]
Sn	0.1 m KHCO_3_	Formate	94.9	5	−1.55 vs. NHE	/	[69]
Sn NPs	1 m KHCO_3_	Formate	94	140	−3.5	550 h	[205]
Sn(S)/Au	0.1 m KHCO_3_	Formate	93.3	55	−0.75	40 h	[197]
Sn/CN	0.1 m KHCO_3_	Formate	96	3.8	−0.9	10 h	[206]
Sn/graphene	0.1 m NaHCO_3_	Formate	89	21.1	−1.15	50 h	[23]
Sn/rGO800	0.1 m KHCO_3_	Formate	98	9.9	−0.82	30 min	[207]
SnO_2_ NPs	1 m KHCO_3_	Formate	64	440	−1.21	na	[208]
SnO_2_ porous NWs	0.1 m NaHCO_3_	Formate	80	4.8	−0.8	15 h	[24]
SnO_2_/Bi_2_O_3_	0.1 m KHCO_3_	Formate	90	5	−1	12 h	[175]
SnO_2_/CC	0.5 m NaHCO_3_	Formate	87	45	−0.88	24 h	[209]
SnO2‐anod	0.1 m KHCO_3_	Formate	95	10.2	−0.97	5 h	[210]
Sulfur‐doped indium	0.5 m KHCO_3_	Formate	93	58.9	−0.98	10 h	[211]
Sulphide derived‐Bi	0.5 m NaHCO_3_	Formate	84	5	−0.75	24 h	[92]
Surface activated Bi NP	0.5 m NaHCO_3_	CO	96.1	15.6	−2.0 V (vs. Ag/AgCl)	2 h	[65]
TA‐Pb	0.1 m KHCO_3_	Formate	96.4	1	−0.92	60 h	[212]

**TABLE 5:** The performance of recently reported p‐block metal‐based electrocatalysts for NRR.

Catalyst	Electrolyte	Reaction	Product	Faradaic Efficiency (%)	NH_3_ yield	Potential V vs RHE	Longevity	Refs.
Activated Bi_2_Te_3_ NPs/C	0.1 m KOH	NRR	NH_3_	7.9	3.9 µg cm^−2^ h^−1^	−0.4	10 h	[177]
Al‐Co_3_O_4_/NF	0.1 m KOH	NRR	NH_3_	5.25	∼3.97 µg cm^−2^ h^−1^	−0.2	20 h	[144]
Bi nanosheet array	0.1 m HCl	NRR	NH_3_	10.26	4.21 µg cm^−2^ h^−1^	−0.5	6 cycles	[51]
Bi nanosheets	0.1 m Na_2_SO_4_	NRR	NH_3_	10.46	13.23 mg g^−1^ h^−1^	−0.8	6 cycles	[51]
Bi ND/CP	0.1 m HCl	NRR	NH_3_	10.8	25.86 mg g^−1^ h^−1^	−0.6	6 cycles	[213]
Bi NPs	0.1 m Na_2_SO_4_	NRR	NH_3_	12.11	3.25 µg cm^−2^ h^−1^	−0.6	10 cycles	[49]
Bi_4_O_5_I_2_‐OH	0.1 m Na_2_SO_4_	NRR	NH_3_	32.4	20.44 mg g^−1^ h^−1^	−0.1	12 h	[22]
Bismuth nanoplates	0.2 m Na_2_SO_4_	NRR	NH_3_	11.68	5.45 mg g^−1^ h^−1^	−0.6	24 h	[214]
BiVO_4_	0.2 m Na_2_SO_4_	NRR	NH_3_	10.04	8.6 mg g^−1^ h^−1^	−0.5	4 cycles	[214]
Flower‐like β‐Bi_2_O_3_	0.1 m Na_2_SO_4_	NRR	NH_3_	4.3	19.92 mg g^−1^ h^−1^	−0.8	20 h	[215]
Ga SA/a‐TiO_2_	0.1 m Na_2_SO_4_	NRR	NH_3_	48.64	24.47 mg g^−1^ h^−1^	−0.1	24 h	[29]
Heterostructure catalyst Sn–SnO_2_/NC	0.1 m Na_2_SO_4_	NRR	NH_4_	41.3	30.3 µg mg^−1^ h^−1^	−0.05	12 h	[216]
Indium‐tin oxide glass	0.5 m LiClO_4_	NRR	NH_3_	6.17	6.49 µg g^−1^ h^−1^	−0.4	24 h	[217]
In‐MOF	1 m KOH	NRR	NH_3_	14.98	79.2 mg g^−1^ h^−1^	0.55	12 h	[218]
Nanoporous NiSb alloy	0.1 m HCl	NRR	NH_3_	48	56.9 mg g^−1^ h^−1^	−0.2	5 cycles	[28]
PdPb/C	0.1 m HCl	NRR	NH_3_	1.19	37.68 mg g^−1^ h^−1^	−0.05	10 h	[219]
Sb1/a‐MoO_3_	0.5 m Na_2_SO_4_	NORR	NH_3_	91.7	∼4.66 mg cm^−2^ h^−1^	−0.6	30 h	[103]
Sb_2_S_3_	0.5 m LiClO_4_	NRR	NH_3_	24.1	33.4 mg g^−1^ h^−1^	−0.3	15 h	[220]
Sn/SnS_2_ nanosheets	0.1 m PBS	NRR	NH_3_	3.4	23.8 mg g^−1^ h^−1^	−0.8	25 h	[221]
SnS@C	0.1 m Na_2_SO_4_	NRR	NH_3_	14.56	24.33 mg g^−1^ h^−1^	−0.5	18 h	[222]
Ultrafine Sn nanoparticles	0.1 m Na_2_SO_4_	NRR	NH_3_	14.87	17.28 mg g^−1^ h^−1^	−0.4	4 cycles	[223]

**TABLE 6:** The performance of recently reported p‐block metal‐based electrocatalysts for ORR.

Catalyst	Electrolyte	Product	Mass Activity (A mg^−1^)	Specific Activity (mA cm^−2^)	Electron Transfer number	Half‐wave Potential vs RHE	Longevity	Refs.
BiMn_2_O_5_	0.1 m KOH	H_2_O	1.02	0.01	3.9	0.79	/	[143]
Bi−Pd/Cmetallene	0.1 m KOH	H_2_O	11.34@0.9V	8.95@0.9V	3.9	0.927	30k cycles	[148]
FeSn–C2N	0.1 m KOH	H_2_O	0.75	5	3.9‐4.1	0.914	550min	[224]
FeSn/NC	0.1 m KOH	H_2_O	0.92@0.85V	1.22@0.85V	3.92	0.90	700h	[161]
Pd_5_Bi_2_	0.1 m KOH	H_2_O	2.05@0.9V	/	/	0.93	10k cycles	[168]
PdPbHx	0.1 m KOH	H_2_O	1.36@0.95V	1.59@0.95V	3.995	0.958	50k cycles	[158]
Pt_2_In_0.2_Ni_1.8_	0.1 m HClO_4_	H_2_O	0.76@0.9V	1.96@0.9V	/	0.9	16k cycles	[173]
Pt_4.31_Ga	0.1 m HClO_4_	H_2_O	1.89@0.9V	3.28@0.9V	/	/	30k cycles	[26]
SnNC	0.1 m HClO_4_	H_2_O	1.0@0.8V	0.15	4	0.73	9k cycles	[27]

Beyond these conventional electrocatalytic energy conversion applications, urea synthesis via electrocatalytic C‐N coupling has emerged as a promising and sustainable alternative to traditional industrial urea production [31, 84]. In particular, *p*‐block metal‐based catalysts have recently attracted increasing attention in this area due to their excellent performance in CO_2_RR and nitrogen or nitrate reduction reactions, enabling efficient C‐N coupling through the integration of these two reduction processes [31]. Although this research field is still at an early stage, it is reasonable to anticipate that p‐block metal‐based catalysts for electrocatalytic urea synthesis will become an important and rapidly growing area in the coming years. Several representative and significant studies have been summarized in Table 7.

**TABLE 7:** The performance of recently reported p‐block metal‐based electrocatalysts for electrocatalytic urea synthesis.

Catalyst	C/N sources	Electrolyte	Cell type	Potential V vs. RHE	Urea yield rate	Faradaic Efficiency(%)	Longevity	Ref.
SnO_2_CuCo	CO_2_, NO_3_^−^	0.1 m KNO_3_	H‐cell	−0.4	2701.2± 99.1µmol h^−1^gcat ^−1^	51	10 cycles	[225]
Sn_2_Cu	CO_2_, NO_3_^−^	0.4 m KNO_3_	Flow cell	−0.52	72.6 mmolh^−1^ gcat^−1^	41.3	60h	[226]
0.06Bi_2_O_3_‐In_2_O_3_/Cu_2_O	CO_2_, NO_3_^−^	0.1 m KNO_3_	H‐cell	−0.9	978.68 µgh^−1^ mgcat^−1^	39.24	6h	[227]
Bi_SA_/C	CO_2_, NO	0.1 m KHCO_3_	MEA cell	−0.7	86.5 mmol h^−1^g^−1^	52.1	200h	[228]
Bi‐In_2_O_3_‐O_v_	CO_2_, NO_3_^−^	0.1 m KNO_3_	H‐cell	−0.4	2380 µgh^−1^ mgcat^−1^	80.2	120h	[229]
Ga/Y‐CNP	CO_2_, NO_3_^−^	0.05 m KNO_3_, 0.1 m KHCO_3_	H‐cell	−1.4	41.9 mmol h^−1^g^−1^	22.1	21 cycles	[230]
O‐Bi_M_/CuO_X_	CO_2_, NO_3_^−^	0.2 m KHCO_3_, 0.1 m KNO_3_	H‐cell	−0.6	2180.3 µgh^−1^ mgcat^−1^	23.5	10 cycles	[31]

Bi, Sn, In, and Pb consistently demonstrate high Faradaic efficiencies for CO_2_RR to formate (> 90%), while Bi uniquely supports NRR through favorable 6p–2p orbital overlap with nitrogen. Additionally, these metals exhibit high electivity for the two‐electron ORR mechanism, driven by a weak ^*^OOH adsorption that prevents the breaking of O─O bonds. These characteristics highlight the fundamental advantages that p‐block metals possess when considering reaction selectivity despite their intrinsic inferior catalytic activity to PGMs. Although Sb, Sn, and Bi are extensively studied, it should not be forgotten that the impact of Al, Ga, and Ge. Their ability to form strong Lewis acids makes them promising candidates for electrosynthesis. Addressing the toxicity of heavy p‐block elements like Pb and Sb will prove challenging despite the draw of their electrocatalytic properties. Simply using these elements in catalytic structures could result in toxic byproducts due to dissolution under operating conditions [231]. Ideally, if leaching occurs, stabilizing the structure to form a less toxic oxidation state (e.g., Sb^5^^+^) is preferred. Enhanced stability is achieved through several strategies, including the use of high‐density anchoring sites, encapsulation within protective layers, or fine‐tuning of the coordination environment. SACs are especially challenging due to their covalent dominant nature [232]. DACs and TACs might also provide a suitable solution as their interconnected systems create strong binding to fix the atoms in place and prevent toxic oxidation states. Recently, a SAC was modified by high‐density carbon, which provided a cost‐effective solution to this leaching challenge [233]. However, the synthesis of these dual and tri systems is challenging, leading to unintended toxic waste. Yet progress has been made in understanding the synthesis mechanisms [234]. Similar approaches to alloy and doped materials could also benefit. Another interesting approach is to take advantage of the P‐block metals' Lewis acid nature and incorporate them into polymer networks or protective coatings [235]. Similar supporting structures and designs would advance the field considerably.

### 6.2 Design Strategies and Emerging Directions

Several design strategies have enabled significant performance improvements in creating a competitive p‐block metal‐based catalyst. SACs maximized atom utilization and lowered costs while enabling tailored electronic environments, but their rapid degradation and poor scalability limited practical application. Doped catalysts improved stability and electronic flexibility, yet encountered significant scaling challenges. Consequently, research focus moved toward sophisticated architectures like multi‐atom active sites (DACs and TACs), where neighboring atoms cooperated to facilitate reaction pathways unavailable to single‐metal catalysts. The ORR reaction specifically would benefit from DACs and TACs. Dual and tri‐atom configurations would enable cooperative adsorption and activation of O_2_ and oxygenated intermediates. Computational models have demonstrated that P‐block supporting dual atom sites provide exceptional OER and ORR function and stability [236]. Importantly, the performance cannot be fully rationalized using traditional d‐band theory, highlighting the unique electronic characteristics of these multi‐atom p‐block sites. Although the design of optimal pairings and supporting structures is still in its early stages, such DACs and TACs offer promising avenues for developing bifunctional catalysts that approach the performance of noble metals. Furthermore, alloys and intermetallics, particularly those combining p‐block and transition metals, exploited synergistic effects to decouple adsorption energies and enhance catalytic efficiency. Heterostructures and 2D materials introduce new functionalities, such as built‐in electric fields, high charge transfer, and strain‐modulated band structures. Together, these design strategies provide a rich toolbox for overcoming the inherent limitations of pure p‐block catalysts. A challenge in compiling multiple catalysts from the literature is the varying standards of benchmarking. The duration and conditions of stability testing vary widely, often optimized to showcase a material's best performance rather than provide a comprehensive assessment of its practical viability. Confusion is compounded as different reactions can have differing expected benchmarks. However, the nature of discovery does not always lead to the ultra‐durable and high‐performance catalyst that is revolutionary. Understanding the performance of the quick‐degrading and low‐performance catalyst also provides valuable insight into designing the next high‐performance material. To address this, we propose a two‐tiered benchmarking framework that distinguishes between laboratory‐scale discovery research and industrially transitioning technologies.

For fundamental catalyst discovery, we recommend the following minimum standards:

Chronopotentiometry/Chronoamperometry: A minimum duration of 10–20 h at constant current density should be reported. This timeframe is sufficient to capture initial degradation mechanisms while remaining achievable within typical laboratory workflows. Many studies currently report only 1–2 h of stability data, which often misses early‐stage degradation processes. The recommended current densities are reaction‐specific: 10 mA cm^−2^ (geometric area) for HER and OER, and 5–10 mA cm^−2^ for CO_2_RR and NRR, where mass transport limitations typically restrict achievable current densities.

Cyclic Voltammetry: Accelerated stability testing via CV should include a minimum of 1000 cycles at scan rates of 50–100 mV s^−^^1^. This provides insight into electrochemical surface area changes and electrochemical stability under dynamic conditions.

Characterization Requirements: Pre and post stability characterization should include microscopy (SEM/TEM) to assess morphological changes, and surface/bulk analysis (XPS or XRD) to track chemical state evolution. Faradaic efficiency should be measured at multiple timepoints throughout stability testing (beginning, middle, and end) rather than only at initial conditions.

Reporting Standards: In addition to geometric current density, studies should report intrinsic activity metrics such as turnover frequency (TOF) or mass activity. Control experiments with benchmark materials (e.g., Pt/C for HER, IrO_2_/RuO_2_ for OER, Ag for CO_2_RR) and support‐only controls are essential for contextualizing performance.

For catalysts approaching practical application, more stringent protocols are necessary:

Extended Stability Testing: Chronopotentiometry should be conducted for 100–1000 h, representing an intermediate milestone toward commercial deployment. The U.S. Department of Energy suggests over 5 000 h for commercial water electrolysers, making 100–1000 h a reasonable pre‐commercial target.

Industrially‐Relevant Current Densities: Testing should be performed at elevated current densities that reflect economic viability: 100–500 mA cm^−2^ for HER, 100–300 mA cm^−2^ for OER, 100–200 mA cm^−2^ for CO_2_RR, and 50–100 mA cm^−2^ for ORR. These values align with current densities employed in industrial electrochemical processes.

Performance Degradation Metrics: A maximum overpotential increase of 10% over the testing duration should be targeted. Greater degradation indicates fundamental stability limitations that must be addressed before scale‐up.

Realistic Operating Conditions: Testing should be conducted in flow cells or other industrially relevant geometries, rather than static H‐cells. Where applicable, elevated temperatures (40°C–80°C) and real feedstocks (industrial CO_2_ sources, non‐ultrapure water) should be employed to assess performance under practical conditions.

These proposed standards provide a framework for more rigorous and comparable evaluation of p‐block electrocatalysts. Adoption of such protocols would accelerate the field's transition from fundamental discovery to practical implementation by establishing clearer performance milestones and enabling more meaningful cross‐study comparisons.

### 6.3 Challenges and Practical Barriers

Despite these advances, several obstacles limit industrial implementation. Many p‐block catalysts degrade rapidly under electrochemical conditions through leaching, oxidation, or structural collapse. Bi‐based materials and other catalysts often lose activity after only a few hours. The dynamic restructuring of active sites led to reproducibility problems in SACs and doped catalysts, whereas alloy fabrication generally involved laborious methods and was restricted to small batches. Moreover, most reported catalysts achieve peak Faradaic efficiency in narrow voltage windows, which is unsuitable for industrial operation. Benchmarking protocols for stability, durability, and scalability are still lacking, hindering fair comparisons across studies. Addressing these issues is critical for translating laboratory performance into real‐world systems. Many of these limitations of SACs are derived from a limited understanding of atomistic interactions and how deactivation progresses. Comprehending these mechanisms will improve countermeasures and introduce a new area of research. However, the three‐phase boundary makes modelling these interactions in DFT challenging.

### 6.4 Outlook

The next stage in p‐block electrocatalyst development requires shifting from trial‐and‐error approaches toward data‐driven discovery. High‐throughput computation, machine learning, and combinatorial synthesis can accelerate the identification of descriptors that govern activity and selectivity, particularly for underexplored heavier elements such as Sb and Pb. Mechanistic insights also demand greater attention: the role of p‐band centers, scaling relation deviations, and morphology‐driven site creation remains underdeveloped but could provide pathways to break long‐standing activity/selectivity trade‐offs. In recent years, a boom in implicit solvation and electric double‐layer models has been developed. These designs better represent the environmental factors and replicate real conditions. Furthermore, advanced computational or simulative models should explicitly account for degradation mechanisms, such as atom migration, dissolution, surface reconstruction, or poisoning, to better predict the lifetime and durability of the catalyst. Ultimately, environmentally friendly and scalable synthesis strategies became crucial for moving p‐block catalysts from lab‐scale development to commercial deployment. By combining mechanistic understanding with rational design and scalable production, p‐block metals could form the foundation of the next generation of sustainable electrocatalysts.

## Conflicts of Interest

The authors declare no conflicts of interest.

## Data Availability

The authors have nothing to report.
